# Supplementary material for: Emergence of cooperation promoted by higher-order strategy updates
Source: PLoS Comput Biol. 2025 Aug 4;21(8):e1012891. doi: 10.1371/journal.pcbi.1012891 (PMC12321138; doi:10.1371/journal.pcbi.1012891)
Supplement: S1 Text — (ZIP) [file pcbi.1012891.s001.zip › S1_Text/S1 Text_revise_nomark.pdf]

Supporting Information for

Emergence of cooperation promoted by higher-order strategy updates

Dini Wang, Peng Yi, Yiguang Hong, Jie Chen and Gang Yan

This PDF file includes:

Roadmap

Mathematical Derivations and Supplementary Results

Figure S1

SI References

Here is a roadmap for the Supporting Information versus the main text:

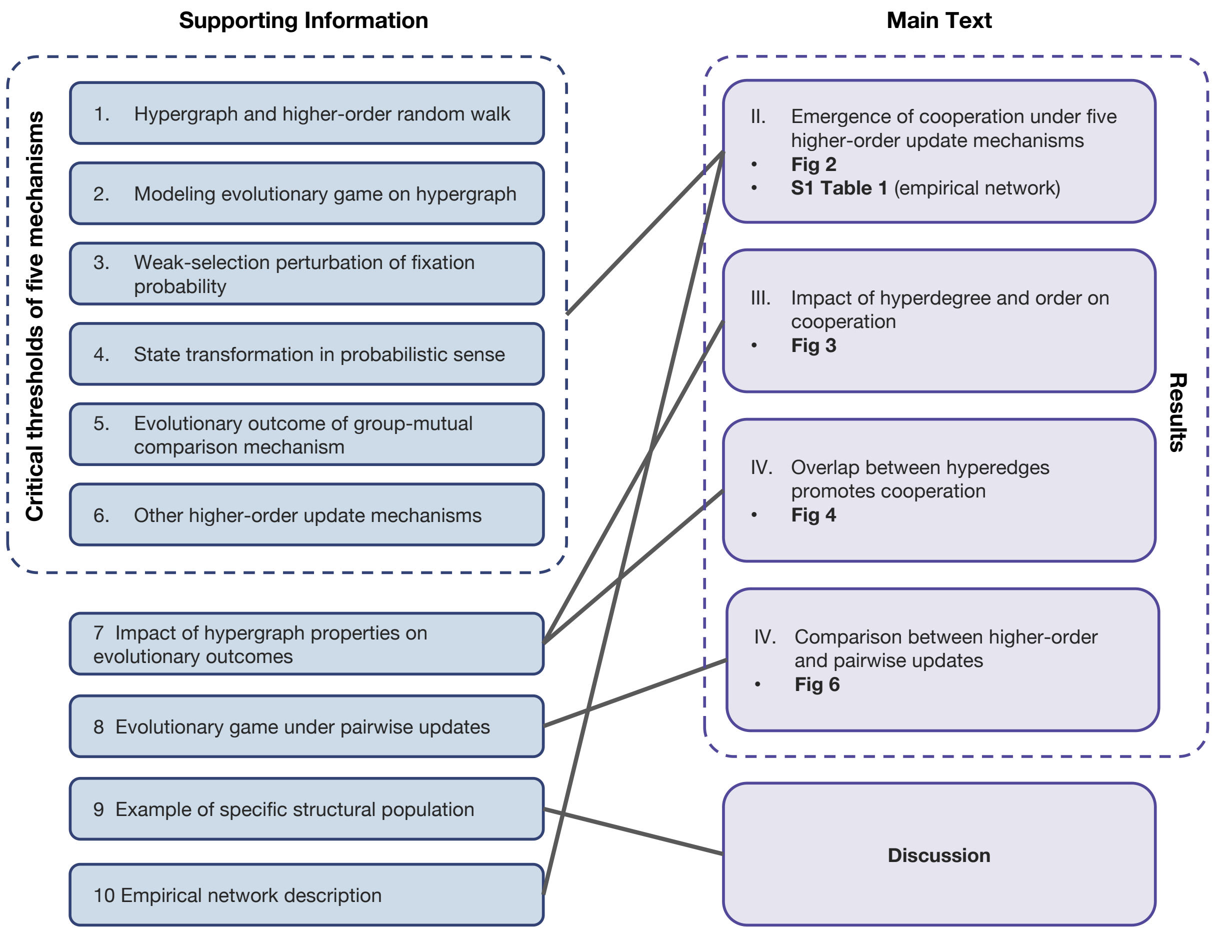

Contents

1 Hypergraph and higher-order random walk **2**

1.1 Hypergraph . . . 2

1.2 Higher-order random walk . . . 2

1.2.1 Self-loop disallowed higher-order random walk . . . 2

1.2.2 Self-loop allowed higher-order random walk . . . 3

1.2.3 Conjoint higher-order random walk . . . 3

2 Modeling evolutionary game on hypergraph **3**

2.1 Public goods game on hypergraph . . . 3

2.2 Strategy update . . . 4

3 Weak-selection perturbation of fixation probability **5**

3.1 Definition of fixation probability . . . 5

3.2 Instantaneous transition under weak selection . . . 5

4 State transformation in probabilistic sense **6**

5 Evolutionary outcome of group-mutual comparison mechanism **7**

5.1 Coalescing random walks . . . 7

5.2 Eliminating infinite summation . . . 11

5.3 Critical synergy factor . . . 12

6 Other higher-order update mechanisms **13**

6.1 Higher-order death-birth mechanism . . . 13

6.1.1 Fixation probability under weak selection . . . 13

6.1.2 Coalescence process in probabilistic sense . . . 15

6.1.3 Critical synergy factor . . . 16

6.2 Higher-order imitation mechanism . . . 16

6.2.1 Fixation probability under weak selection . . . 16

6.2.2 Coalescence process in probabilistic sense . . . 18

6.2.3 Critical synergy factor . . . 19

6.3 Group-inner comparison mechanism . . . 20

6.3.1 Fixation probability under weak selection . . . 20

6.3.2 Coalescence process in probabilistic sense . . . 21

6.3.3 Critical synergy factor . . . 22

6.4 Higher-order pair-comparison mechanism . . . 22

6.4.1 Fixation probability under weak selection . . . 22

6.4.2 Coalescence process in probabilistic sense . . . 23

6.4.3 Critical synergy factor . . . 25

7 Impact of hypergraph properties on evolutionary outcomes 25

7.1 Hyperdegree and order . . . 26

7.2 Hyperdegree heterogeneity . . . 27

7.3 Order heterogeneity . . . 28

7.4 Overlap strength . . . 29

8 Evolutionary game under pairwise updates 30

8.1 Death-birth mechanism . . . 30

8.2 Pair-comparison mechanism . . . 30

9 Example of specific structural population 31

9.1 Isolated population . . . 31

9.2 Overlapped population . . . 31

10 Empirical network description 31

1 Hypergraph and higher-order random walk

1.1 Hypergraph

Population structure can be effectively modeled using a connected hypergraph, $\mathcal{H}(\mathcal{N},\mathcal{E})$, where $\mathcal{N}$ is a set of N nodes, and $\mathcal{E}\subseteq\{e|e\subseteq\mathcal{N}\}$ is a set of E hyperedges. Such a hypergraph can be completely described by an incidence matrix $B_{N\times E}$, with entries $b(i,e)=1$ if node i belongs to hyperedge e (*i.e.*, $i\in e$), and 0 otherwise. Moreover, there are two basic properties of the hypergraph: hyperdegree and order. Specifically, the hyperdegree of a node $i\in\mathcal{N}$ is the number of hyperedges that node i belongs to, formally defined as $k_i=\sum_{e\in\mathcal{E}} b(i,e)$; and the order of a hyperedge $e\in\mathcal{E}$ is the number of nodes inside the hyperedge e, calculated as $g_e=\sum_{i\in\mathcal{N}} b(i,e)$. Notably, each hyperedge must connect at least two nodes, thus $g_e\geq 2$ for each $e\in\mathcal{E}$.

1.2 Higher-order random walk

For the convenience of analyzing the evolutionary dynamics on the hypergraph, we establish the higher-order random walk to model a walker moving from a position (node) to another according to its local structural information. To match with the two-stage selection of higher-order updates, one step of the higher-order random walk on the hypergraph unfolds into two stages: a walker firstly selects an adjacent hyperedge, and whereafter selects a target node in the chosen hyperedge to move to the location of the node. The selection in both stages is uniformly at random. Here we consider both self-loop disallowed and self-loop allowed versions of the higher-order random walk, as well as their conjoint pattern.

1.2.1 Self-loop disallowed higher-order random walk

We begin with the self-loop disallowed higher-order random walk (SDHRW), which forbids the walker to keep the position unchanged in one hop. For such a random walk on the hypergraph $\mathcal{H}$ from node i to j, the probability of selecting the adjacent hyperedge $e\in\mathcal{E}$ is given by

$$q(i,e)=\frac{b(i,e)}{k_i}. \tag{S1}$$

Then within the hyperedge e, the probability of selecting the target node j $(j\neq i)$ is given by

$$\widetilde{q}(e,j)=\frac{b(j,e)}{g_e-1}. \tag{S2}$$

Therefore, combining these two stages produces the overall probability of a SDHRW from node i to j as

$$\widetilde{p}_{ij}=\begin{cases}\sum_{e\in\mathcal{E}} q(i,e)\widetilde{q}(e,j) & j\neq i\\ 0 & j=i\end{cases}. \tag{S3}$$

The one-step transition probability matrix of the SDHRW on $\mathcal{H}$ is defined as $\widetilde{P}=\left[\widetilde{p}_{ij}\right]_{N\times N}$. Hence, the n-step analogue is denoted by $\widetilde{P}^n$, whose (i,j)-th entry, $\widetilde{p}_{ij}^{(n)}$, represents the probability that n steps from node i terminate at node j with self-loops not allowed. Furthermore, in the limit of n approaching infinity, the SDHRW on $\mathcal{H}$ has a unique stationary distribution $\{\pi_i\}_{i\in\mathcal{N}}$, where each element satisfies $\pi_i=k_i/\sum_{j\in\mathcal{N}} k_j$ [1]. Thus, for each $i,j\in\mathcal{N}$, $\lim_{n\to\infty}\widetilde{p}_{ij}^{(n)}=\pi_j$. There is a reversibility property of the SDHRW on $\mathcal{H}$, namely, $\pi_i\widetilde{p}_{ij}^{(n)}=\pi_j\widetilde{p}_{ji}^{(n)}$ for all $i,j\in\mathcal{N}$.

1.2.2 Self-loop allowed higher-order random walk

In what follows, we consider the self-loop allowed higher-order random walk (SAHRW). Akin to the case with no self-loops, the SAHRW includes two stages (*i.e.*, selecting an adjacent hyperedge and selecting a node in the hyperedge), with the distinction that the walker is permitted to remain at the current position in the SAHRW, whereas in the SDHRW it is not. For a SAHRW on the hypergraph $\mathcal{H}$, the walker located in node $i \in \mathcal{N}$ firstly selects a neighboring hyperedge $e \in \mathcal{E}$ also with the probability $q(i,e)$. Subsequently, divergent from SDHRW, the walker can select any node $j \in e$ (including $j = i$) as the next destination, and the probability of the node selection in the SAHRW is

$$\widehat{q}(e,j) = \frac{b(j,e)}{g_e}. \tag{S4}$$

The overall transition probability from node i to j combining both stages can be calculated as

$$\widehat{p}_{ij} = \sum_{e \in \mathcal{E}} q(i,e)\widehat{q}(e,j). \tag{S5}$$

Accordingly, one-step transition probability matrix of the SAHRW is denoted as $\widehat{P} = \left[\widehat{p}_{ij}\right]_{N\times N}$, and the n-step matrix $\widehat{P}^n$ is composed of the n-step probability $\widehat{p}_{ij}^{(n)}$ from the node i to j on the hypergraph $\mathcal{H}$ with the SAHRW. Also when n is substantially large, the system is fixed as the stationary distribution $\{\pi_i\}_{i\in\mathcal{N}}$ in the SAHRW, which is identical to the SDHRW. In addition, the SAHRW on $\mathcal{H}$ is also reversible, that is $\pi_i\widehat{p}_{ij}^{(n)} = \pi_j\widehat{p}_{ji}^{(n)}$ for all $i, j \in \mathcal{N}$.

1.2.3 Conjoint higher-order random walk

Based on the two versions of the higher-order random walk, we introduce the conjoint higher-order random walk (CHRW), where the walker moves n steps with the SDHRW followed by m steps with the SAHRW, hence also nominated as (n,m)-step higher-order random walk. The probability of such an (n,m)-step higher-order random walk starting at node i and concluding at node j is

$$p_{ij}^{(n,m)} = \sum_{x\in\mathcal{N}} \widetilde{p}_{ix}^{(n)}\widehat{p}_{xj}^{(m)}. \tag{S6}$$

For any function h_i defined on the hypergraph $\mathcal{H}$, we use a shorthand notation:

$$h_i^{(n,m)} = \sum_{j\in\mathcal{N}} p_{ij}^{(n,m)} h_j. \tag{S7}$$

Here $h_i^{(n,m)}$ symbolizes the expected value of h_j, where j is the terminus of an (n,m)-step random walk originating from i.

2 Modeling evolutionary game on hypergraph

2.1 Public goods game on hypergraph

For the public goods game (PGG) on the hypergraph, the state of the evolutionary dynamics can be described as a binary vector $\mathbf{s} = [s_i]_{i\in\mathcal{N}} \in \{0,1\}^N$, where 0 and 1 correspond to defection and cooperation respectively. Each individual engages in the public goods game (PGG) within the hyperedges it belongs to, and the payoff of the individual i playing the PGG in

the hyperedge e is

$$u_{i,e}(\mathbf{s}) = \sum_{j \in \mathcal{N}} b(i,e)b(j,e)s_j \cdot r - b(i,e)s_i. \tag{S8}$$

where r is the order-normalized synergy factor of the PGG. All the payoffs of the games the individual participates in are averaged to obtain the individual's payoff as

$$u_i(\mathbf{s}) = \frac{\sum_{e \in \mathcal{E}} u_{i,e}(\mathbf{s})}{k_i} = \sum_{e \in \mathcal{E}} \sum_{j \in \mathcal{N}} \frac{b(i,e)b(j,e)}{k_i} s_j \cdot r - s_i. \tag{S9}$$

The fitness of i is given by $f_i(\mathbf{s}) = 1 + \delta u_i(\mathbf{s})$, where $\delta > 0$ quantifies the strength of selection. Here we mainly focus on weak selection, that is $\delta \to 0$. From the group perspective, the fitness of a hyperedge e is represented by the average of all the members' payoffs in the hyperedge, calculated as $F_e(\mathbf{s}) = \sum_{i \in \mathcal{N}} \widehat{q}(e,i) f_i(\mathbf{s})$.

2.2 Strategy update

The specific process of a strategy update is essentially that a randomly chosen node takes the two-stage selection of a target node for imitation or comparison. To take the group-mutual comparison (GMC) as an example, we quantitatively describe the probability of the state transition in a strategy update. Firstly, under GMC mechanism and the state $\mathbf{s}$, a to-be-updated node i selects a hyperedge e it belongs to with the probability

$$\Pr\left[i \to e\right](\mathbf{s}) = \frac{b(i,e)F_e(\mathbf{s})}{\sum_{\alpha \in \mathcal{E}} b(i,\alpha)F_\alpha(\mathbf{s})}. \tag{S10}$$

Secondly, the node i selects a neighbor j ($j \neq i$) in the hyperedge e uniformly at random. Under the group selection of e, the conditional probability of this individual selection is

$$\Pr\left[i \to j | i \to e\right](\mathbf{s}) = \frac{b(j,e)}{g_e - 1}. \tag{S11}$$

Since the path from i to j may traverse several hyperedges beyond e, we derive the overall probability of j imitating i's strategy under GMC mechanism as

$$\begin{aligned}
\Pr\left[i \to j\right](\mathbf{s}) &= \sum_{e \in \mathcal{E}} \Pr\left[i \to e\right](\mathbf{s}) \cdot \Pr\left[i \to j | i \to e\right](\mathbf{s}) \\
&= \sum_{e \in \mathcal{E}} \frac{\frac{b(i,e)}{k_i} \sum_{j \in \mathcal{N}} \widehat{q}(e,j) f_j(\mathbf{s})}{\sum_{\alpha \in \mathcal{E}} \frac{b(i,\alpha)}{k_i} \sum_{y \in \mathcal{N}} \widehat{q}(\alpha,y) f_y(\mathbf{s})} \cdot \frac{b(j,e)}{g_e - 1} \\
&= \sum_{e \in \mathcal{E}} \frac{q(i,e) \sum_{j \in \mathcal{N}} \widehat{q}(e,j) f_j(\mathbf{s})}{\sum_{\alpha \in \mathcal{E}} q(i,\alpha) \sum_{y \in \mathcal{N}} \widehat{q}(\alpha,y) f_y(\mathbf{s})} \cdot \widetilde{q}(e,j)
\end{aligned} \tag{S12}$$

For the focal node i, all the possibilities that i imitates its immediate neighbor j add up to 1, according to Eq. [S12]. In mathematical terms, $\sum_{j \in \mathcal{N}, j \neq i} \Pr\left[i \to j\right](\mathbf{s}) = 1$.

3 Weak-selection perturbation of fixation probability

3.1 Definition of fixation probability

After sufficient rounds of updates, the population will ultimately converge to an absorbing state of either all cooperators or all defectors if no mutations occur in the evolutionary process [2]. Here we focus on the probability that the system fixes at the cooperating state. To capture the behavioral dynamics, we establish a continuous-time Markov chain $\mathbf{s}^v(t) = (s_1^v(t), s_2^v(t), \dots, s_N^v(t))'$ for $t \in [0, \infty)$, where each element represent the probability that the node serves as a cooperator at time t with the first mutant of the node v. Hence, $s_v^v(0) = 1$ for the node u whereas $s_i^v(0) = 0$ for other nodes $i \in \mathcal{N} \backslash \{v\}$.

On the initial configuration v, define the fixation probability of cooperation, denoted as ρ_{C}^v, as the reproductive value weighted frequency of cooperators in the sense of expectation until the system reaches the absorbing state, where the reproductive value of the node is numerically equal to the stationary probability of the higher-order random walk on the hypergraph.

$$\rho_{\mathrm{C}}^v := \lim_{t \to \infty} \mathbb{E}\left[\sum_{i \in \mathcal{N}} \pi_i s_i^v(t)\right] \tag{S13}$$

where $\mathbb{E}[\cdot]$ represents the expectation of the formula in the square brackets.

Consider that the initial state is $\phi\left(\mathbf{s}^v(0)\right) = \pi_v$. Then we apply the fundamental formula of calculus to the definition of fixation probability to derive as

$$\rho_{\mathrm{C}}^v = \pi_v + \int_0^\infty \mathbb{E}\left[\sum_{i \in \mathcal{N}} \pi_i \cdot \frac{ds_i^v(t)}{dt}\right] dt. \tag{S14}$$

Since the initial mutant arises at a random node with Poisson rate 1, the fixation probability of cooperation is the average on all the probable initial configurations, calculated as

$$\rho_{\mathrm{C}} = \frac{1}{N} + \int_0^\infty \mathbb{E}\left[\sum_{i,v \in \mathcal{N}} \pi_i \cdot \frac{ds_i^v(t)}{dt}\right] dt. \tag{S15}$$

3.2 Instantaneous transition under weak selection

Next, we elaborate on the instantaneous change of the individual state under weak selection to unfold the fixation probability of Eq. [S15]. For a single node j, its state either remains unchanged or copies the state of the selected node i within an instantaneous time interval. Hence the continuous-time evolution can be formalized as [3]

$$s_j^v(t + \Delta t) = \begin{cases} s_i^v(t) & \text{with rate } \left[\Pr[j \to i](\mathbf{s}^v(t))\Delta t + o(\Delta t)\right] \text{ for } i \in \mathcal{N} \\ s_j^v(t) & \text{with rate } \left[1 - \Delta t + o(\Delta t)\right] \end{cases}. \tag{S16}$$

where t describes the time scale and $\Delta t \to 0$ quantifies the substantially small time interval.

Given the system state $\mathbf{s}^v(t)$, the expected state of node j at time $t + \Delta t$ can be described as

$$\mathbb{E}\left[s_j^u(t + \Delta t) \mid \mathbf{s}^v(t)\right] = \sum_{i \in \mathcal{N}} \Pr[j \to i](\mathbf{s}^v(t))\Delta t \cdot s_i^v(t) + \left(1 - \Delta t\right) \cdot s_j^v(t) + o(\Delta t). \tag{S17}$$

We take the expectation of both sides of Eq. [S17], and obtain the formula as

$$\mathbb{E}\left[s_j^v(t+\Delta t)\right] = \mathbb{E}\left[s_j^v(t)\right] + \mathbb{E}\left[\sum_{i\in\mathcal{N}} \Pr[j \to i](\mathbf{s}^v(t)) \cdot s_i^v(t) - s_j^v(t)\right] \cdot \Delta t + o(\Delta t). \tag{S18}$$

Due to Δt approaching to 0, we can derive the instantaneous change of the node j's state as

$$\lim_{\Delta t\to 0} \mathbb{E}\left[\frac{s_j^v(t+\Delta t) - s_j^v(t)}{\Delta t}\right] = \sum_{i\in\mathcal{N}} \Pr[j \to i]\left(\mathbf{s}^v(t)\right) \cdot s_i^v(t) - s_j^v(t). \tag{S19}$$

Furthermore, the instantaneous change of the node's state is weighted by the corresponding stationary probability to get the instantaneous change of the whole system's state.

$$\begin{aligned}
\sum_{j\in\mathcal{N}} \pi_j \cdot \frac{ds_j^v(t)}{dt} &= \sum_{j\in\mathcal{N}} \pi_j \left(\sum_{i\in\mathcal{N}} \Pr[j \to i]\left(\mathbf{s}^v(t)\right) \cdot s_i^v(t) - s_j^v(t)\right) \\
&= \sum_{j\in\mathcal{N}} \pi_j \left(\sum_{i\in\mathcal{N}} s_i^v(t) \sum_{e\in\mathcal{E}} \frac{q(j,e)\sum_{y\in\mathcal{N}} \widehat{q}(e,y) f_y(\mathbf{s}^v(t))}{\sum_{\alpha\in\mathcal{E}} q(j,\alpha)\sum_{z\in\mathcal{N}} \widehat{q}(\alpha,z) f_z(\mathbf{s}^v(t))} \cdot \widetilde{q}(e,i) - s_j^v(t)\right) \\
&= \delta \cdot \left[\sum_{i\in\mathcal{N}} s_i^v(t) \sum_{j\in\mathcal{N}} \pi_j \sum_{e\in\mathcal{E}} q(j,e)\widetilde{q}(e,i) \left(\sum_{y\in\mathcal{N}} \widehat{q}(e,y) u_y(\mathbf{s}^v(t)) - \sum_{\alpha\in\mathcal{E}} q(j,\alpha) \sum_{z\in\mathcal{N}} \widehat{q}(\alpha,z) u_z(\mathbf{s}^v(t))\right)\right]_{\delta=0} + O(\delta^2) \\
&= \delta \cdot \left[\sum_{i,y\in\mathcal{N}} \sum_{e\in\mathcal{E}} s_i^v(t) \frac{k_i}{\sum_{z\in\mathcal{N}} k_z} \frac{\sum_{j\in\mathcal{N}} b(j,e)}{k_i} \frac{b(i,e)}{g_e} \frac{b(y,e)}{g_e} u_y(\mathbf{s}^v(t)) - \sum_{i,j,z\in\mathcal{N}} \sum_{e\in\mathcal{E}} s_i^v(t)\pi_j \widetilde{p}_{ji} \widehat{p}_{jz} u_z(\mathbf{s}^v(t))\right]_{\delta=0} + O(\delta^2) \\
&= \delta \cdot \left[\sum_{i,y\in\mathcal{N}} s_i^v(t)\pi_i \widehat{p}_{iy} u_y(\mathbf{s}^v(t)) - \sum_{i,j,z\in\mathcal{N}} s_i^v(t)\pi_i \widetilde{p}_{ij} \widehat{p}_{jy} u_z(\mathbf{s}^v(t))\right]_{\delta=0} + O(\delta^2) \\
&= \delta \cdot \left[\sum_{i\in\mathcal{N}} \pi_i s_i^v(t) \left(u_i(\mathbf{s}^v(t))^{(0,1)} - u_i(\mathbf{s}^v(t))^{(1,1)}\right)\right]_{\delta=0} + O(\delta^2).
\end{aligned} \tag{S20}$$

where one-order Taylor expansion around $\delta = 0$ is performed.

Substituting Eq. [S20] into the fixation probability [S15], we get the weak-selection perturbation of fixation probability as

$$\begin{aligned}
\rho_{\mathrm{C}} =& \frac{1}{N} + \frac{\delta}{N}\int_0^\infty \mathbb{E}\left[-\left(\sum_{i,v\in\mathcal{N}} \pi_i s_i^v(t) s_i^v(t)^{(0,1)} - \sum_{i,v\in\mathcal{N}} \pi_i s_i^v(t) s_i^v(t)^{(1,1)}\right)\right. \\
&\left. + \left(\sum_{i,j,y,v\in\mathcal{N}} \pi_i s_i^v(t) p_{ij}^{(0,1)} \sum_{e\in\mathcal{E}} \frac{b(j,e)b(y,e)}{k_j} s_y^v(t) - \sum_{i,j,y,v\in\mathcal{N}} \pi_i s_i^v(t) p_{ij}^{(1,1)} \sum_{e\in\mathcal{E}} \frac{b(j,e)b(y,e)}{k_j} s_y^v(t)\right) \cdot r\right]_{\delta=0} dt + O(\delta^2).
\end{aligned} \tag{S21}$$

4 State transformation in probabilistic sense

We introduce a probability vector for the dynamical system as $\mathbf{x}^v(t) = (x_1^v(t), x_2^v(t), \ldots, x_N^v(t))'$ for $t \in [0,\infty)$ to match the state vector, where $x_i^v(t) = \Pr(s_i^v(t) = 1)$ indicates the probability of node i being a cooperator at time t on the initial configuration v. Hence, $0 \le x_i^v(t) \le 1$, and $x_i^v(t) = 1$ for cooperator while $x_i^v(t) = 0$ for defector. For an update process, the

state of the hypergraph evolves following the below formula [4, 5]:

$$\begin{aligned} \mathbf{x}^v(t+1) &= \mathbf{P}(\mathbf{s}^v(t)) \cdot \mathbf{s}^v(t) \\ \mathbf{s}^v(t+1) &= \mathcal{R}(\mathbf{x}^v(t+1)) \end{aligned}, \tag{S22}$$

where $\mathbf{P}(\mathbf{s}^v(t))$ is a state transition matrix based on the state $\mathbf{s}^v(t)$, and in the matrix each element $\mathbf{P}(\mathbf{s}^v(t))_{ij}$ is assigned to be the state transition rate from j to i as $\Pr[i \to j](\mathbf{s}^v(t))$. And the operator $\mathcal{R}(\cdot)$ is a realization of the probability vector in the square brackets. In details, $s_i^v(t+1)$ is determined as 1 with the probability of $x_i^v(t+1)$ or 0 with the probability of $1 - x_i^v(t+1)$ for $i \in \mathcal{N}$. Hence, $\mathbf{x}^v(t) = \mathbb{E}(\mathbf{s}^v(t))$ at each time step.

Subsequently, we apply the individual-based mean-field approximation into the neutral drift case to neglect the dynamical correlations between the states of the neighbors, that is

$$\mathbb{E}\left[s_i^v(t)s_j^v(t)\right]_{\delta=0} = \left[x_i^v(t)\right]_{\delta=0} \cdot \left[x_j^v(t)\right]_{\delta=0}. \tag{S23}$$

We then substitute the Eq. [S23] into the weak-selection perturbation of fixation probability [S21], thus yielding the condition that cooperation favors defection (*i.e.*, $\rho_{\mathrm{C}} > 1/N$) as

$$\begin{aligned} &\int_0^\infty \left[\sum_{i,j,y,v\in\mathcal{N}} \pi_i x_i^v(t) \left(p_{ij}^{(0,1)} \sum_{e\in\mathcal{E}} \frac{b(j,e)b(y,e)}{k_j} x_y^v(t) - p_{ij}^{(1,1)} \sum_{e\in\mathcal{E}} \frac{b(j,e)b(y,e)}{k_j} x_y^v(t)\right)\right]_{\delta=0} dt \cdot r \\ &> \int_0^\infty \left[\sum_{i,v\in\mathcal{N}} \pi_i x_i^v(t) \left(x_i^v(t)^{(0,1)} - x_i^v(t)^{(1,1)}\right)\right]_{\delta=0} dt \end{aligned} \tag{S24}$$

5 Evolutionary outcome of group-mutual comparison mechanism

5.1 Coalescing random walks

Here we merely concentrate on the neutral drift (*i.e.*, $\delta = 0$) to unpack the complex intertwinement of the state probability in the fixation probability. To this end, we introduce the coalescing random walk to trace the ancestor backwards in a probabilistic sense. On the neutral drift, the node i copies the state of the node j under group-mutual comparison (GMC) mechanism for any initial mutant v and for any time t with probability

$$\left[\Pr[i \to j](\mathbf{x}^v(t))\right]_{\delta=0} = \widetilde{p}_{ij}. \tag{S25}$$

This reveals an agreement between the higher-order random walk and the selection of the target node.

Regarding the asynchronous update, one-step transition probability of the state with rate $1/N$ can be described as

$$\widetilde{\xi}_{ij} = \begin{cases} 1 - \dfrac{1}{N} & i = j \\ \dfrac{1}{N}\widetilde{p}_{ij} & i \neq j \end{cases}. \tag{S26}$$

The one-step transition matrix of GMC is

$$\widetilde{\Xi} = \frac{1}{N}\widetilde{P} + \left(1 - \frac{1}{N}\right)E, \tag{S27}$$

where E is the identity matrix. Introducing the time scale produces the corresponding matrix $\widetilde{\Xi}(t)$, where each element $\widetilde{\xi}_{ij}(t)$ for $i, j \in \mathcal{N}$ represents the transition probability over t time steps. According to the Eq. [S26], we can simply have $\widetilde{P} = N\widetilde{\Xi} - (N-1)E$, and thus both $\widetilde{\Xi}$ and $\widetilde{P}$ share a common stationary distribution $\{\pi_i\}_{i\in\mathcal{N}}$. Then the reversibility property remains applicable in a continuous-time context, that is $\pi_i\widetilde{\xi}_{ij}(t) = \pi_j\widetilde{\xi}_{ji}(t)$.

The current state of a node can be traced back to the first mutant via higher-order random walks, and hence this process can be expressed as

$$\left[\mathbf{x}^v(t)\right]_{\delta=0} = \widetilde{\Xi}(t) \cdot \left[\mathbf{x}^v(0)\right]_{\delta=0}. \tag{S28}$$

Mathematically, the current state depends on both the initial configuration and the transition probability of neutral drift.

From the node's perspective, the node i's state at time t under the initial configuration v can be traced back to the initial state of the node v. Since the node v's initial state is fixed as 1, *i.e.,* $x_v^v(0) = 1$, the node i's state at time t can be represented as

$$\left[x_i^v(t)\right]_{\delta=0} = \sum_{j\in\mathcal{N}} \widetilde{\xi}_{ij}(t)x_j^v(0) = \widetilde{\xi}_{iv}(t)x_v^v(0) = \widetilde{\xi}_{iv}(t). \tag{S29}$$

Next, Eq. [S29] is substituted into the inequality [S24] to simplify the condition for cooperation by the coalescing random walk. We start with the terms on the right of the inequality [S24], where the fundamental structures are extracted as

$$\begin{aligned}
&\int_0^\infty \sum_{i,v\in\mathcal{N}} \left[\pi_i x_i^v(t) x_i^v(t)^{(n,0)}\right]_{\delta=0} dt \\
=&\int_0^\infty \sum_{i,j,v\in\mathcal{N}} \pi_v \widetilde{\xi}_{vi}(t)\widetilde{p}_{ij}^{(n)}\widetilde{\xi}_{jv}(t)dt \\
=&\int_0^\infty \sum_{i,j,v\in\mathcal{N}} \pi_v \widetilde{\xi}_{vi}(t)\left[(N\widetilde{\Xi} - (N-1)E)^n\right]_{ij}\widetilde{\xi}_{jv}(t)dt \\
=&\int_0^\infty \sum_{i,j,v\in\mathcal{N}} \pi_v \widetilde{\xi}_{vi}(t)\left[C_n^0 N^n \widetilde{\Xi}^n + C_n^1 N^{n-1}(N-1)\widetilde{\Xi}^{n-1} + ... + C_n^n (N-1)^n E\right]_{ij}\widetilde{\xi}_{jv}(t)dt \\
=&\int_0^\infty \sum_{v\in\mathcal{N}} \pi_v \left(C_n^0 N^n \widetilde{\xi}_{vv}(2t+n) + C_n^1 N^{n-1}(N-1)\widetilde{\xi}_{vv}(2t+n-1) + ... + C_n^n (N-1)^n \widetilde{\xi}_{vv}(2t)\right) dt \\
=&\int_0^\infty \sum_{v,i\in\mathcal{N}} \pi_v \widetilde{\xi}_{vi}(2t)\left[C_n^0 N^n \widetilde{\Xi}^n + C_n^1 N^{n-1}(N-1)\widetilde{\Xi}^{n-1} + ... + C_n^n (N-1)^n E\right]_{iv} dt \\
=&\int_0^\infty \sum_{v,i\in\mathcal{N}} \pi_v \widetilde{\xi}_{vi}(2t) p_{iv}^{(n,0)} dt \\
=&\frac{1}{2}\int_0^\infty \sum_{v,i\in\mathcal{N}} \pi_v \widetilde{\xi}_{vi}(t) p_{iv}^{(n,0)} dt
\end{aligned} \tag{S30}$$

where $n = 0, 2$ in the inequality [S24].

As for the terms on the left of the inequality [S24], we show

$$\int_0^\infty \sum_{i,j,y,v\in\mathcal{N}} \pi_i x_i^v(t) p_{ij}^{(n,1)} \sum_{e\in\mathcal{E}} \frac{b(j,e)b(y,e)}{k_j} x_y^v(t)dt = \int_0^\infty \sum_{i,j,v,y\in\mathcal{N}} \pi_v \widetilde{\xi}_{vi}(t) p_{ij}^{(n,1)} \sum_{e\in\mathcal{E}} \frac{b(j,e)b(y,e)}{k_j}\widetilde{\xi}_{yv}(t)dt \tag{S31}$$

where $n = 0, 1$ in the inequality [S24]. To solve the sophisticated terms on the right of Eq. [S31], we introduce some notations for the following approximations.

$$\widetilde{A}_l^{(n_1,m_1,n_2,m_2)} = \sum_{i,j,y,z,v\in\mathcal{N}} \pi_v \widetilde{\xi}_{vi}(t) p_{ij}^{(n_1,m_1)} \sum_{e\in\mathcal{E}} \frac{b(j,e)b(y,e)}{k_j} \widetilde{\xi}_{yz}(t) p_{zv}^{(n_2,m_2)}; \tag{S32}$$

$$\widetilde{A}_r^{(n_1,m_1,n_2,m_2)} = \sum_{i,j,y,z,v\in\mathcal{N}} \pi_v \widetilde{\xi}_{vi}(t) p_{ij}^{(n_1,m_1)} \widetilde{\xi}_{jy}(t) p_{yz}^{(n_2,m_2)} \sum_{e\in\mathcal{E}} \frac{b(z,e)b(v,e)}{k_z}. \tag{S33}$$

The left term of Eq. [S31] to be solved can be represented as $\widetilde{A}_l^{(n,1,0,0)}$. In what follows, $\widetilde{A}_l^{(n,1,0,0)}$ is approximated as $\widetilde{A}_r^{(0,0,n,1)}$ by two steps of validation: $\widetilde{A}_l^{(n,1,0,0)} \approx \widetilde{A}_r^{(n,1,0,0)}$ and $\widetilde{A}_r^{(n,1,0,0)} \approx \widetilde{A}_r^{(0,0,n,1)}$.

Firstly, we validate $\widetilde{A}_l^{(n,m,0,0)} \approx \widetilde{A}_r^{(n,m,0,0)}$ as follows.

$$\begin{aligned}
\widetilde{A}_l^{(n,m,0,0)} &= \sum_{i,j,v,y\in\mathcal{N}, j\neq y} \pi_v \widetilde{\xi}_{vi}(t) p_{ij}^{(n,m)} \sum_{e\in\mathcal{E}} \frac{b(j,e)b(e,y)}{k_j\cdot(g_e-1)}(g_e-1)\cdot\widetilde{\xi}_{yv}(t) + \sum_{i,j,y\in\mathcal{N}} \pi_v \widetilde{\xi}_{vi}(t) p_{ij}^{(n,m)} \sum_{e\in\mathcal{E}} \frac{b(j,e)}{k_j} \widetilde{\xi}_{jv}(t) \\
&\approx \big(\langle g\rangle - 1\big) \sum_{i,j,y,v\in\mathcal{N}} \pi_v \widetilde{\xi}_{vi}(t) p_{ij}^{(n,m)} \widetilde{p}_{jy} \widetilde{\xi}_{yv}(t) + \sum_{i,j,v\in\mathcal{N}} \pi_v \widetilde{\xi}_{vi}(t) p_{ij}^{(n,m)} \widetilde{\xi}_{jv}(t) \\
&= \big(\langle g\rangle - 1\big) \sum_{i,j,y,v\in\mathcal{N}} \pi_v \widetilde{\xi}_{vi}(t) p_{ij}^{(n,m)} \widetilde{\xi}_{jy}(t) \widetilde{p}_{yv} + \sum_{i,j,v\in\mathcal{N}} \pi_v \widetilde{\xi}_{vi}(t) p_{ij}^{(n,m)} \widetilde{\xi}_{jv}(t)
\end{aligned} \tag{S34}$$

$$\begin{aligned}
\widetilde{A}_r^{(n,m,0,0)} &= \sum_{i,j,v,y\in\mathcal{N}, y\neq v} \pi_v \widetilde{\xi}_{vi}(t) p_{ij}^{(n,m)} \widetilde{\xi}_{jy}(t) \sum_{e\in\mathcal{E}} \frac{b(y,e)b(v,e)}{k_y(g_e-1)}\cdot(g_e-1) + \sum_{i,j,v\in\mathcal{N}} \pi_v \widetilde{\xi}_{vi}(t) p_{ij}^{(n,m)} \widetilde{\xi}_{jv}(t) \sum_{e\in\mathcal{E}} \frac{b(v,e)}{k_v} \\
&\approx \big(\langle g\rangle - 1\big) \sum_{i,j,y,v\in\mathcal{N}} \pi_v \widetilde{\xi}_{vi}(t) p_{ij}^{(n,m)} \widetilde{\xi}_{jy}(t) \widetilde{p}_{yv} + \sum_{i,j,v\in\mathcal{N}} \pi_v \widetilde{\xi}_{vi}(t) p_{ij}^{(n,m)} \widetilde{\xi}_{jv}(t)
\end{aligned} \tag{S35}$$

Thus, it is simple to yield that $\widetilde{A}_r^{(n,m,0,0)} \approx A_r^{(0,0,n,m)}$. For solving the left term of [S31], we set $m = 1$, that is $\widetilde{A}_r^{(n,1,0,0)} \approx A_r^{(0,0,n,1)}$.

Secondly, we validate $\widetilde{A}_r^{(n,1,0,0)} \approx \widetilde{A}_r^{(0,0,n,1)}$ by subtracting the equal term, $\widetilde{A}_r^{(n+1,0,0,0)}$ or $\widetilde{A}_r^{(0,0,n+1,0)}$, for approximation.

$$\begin{aligned}
\widetilde{A}_r^{(n,1,0,0)} - \widetilde{A}_r^{(n+1,0,0,0)} =& \sum_{i,j,v,y,z\in\mathcal{N}} \pi_v \widetilde{\xi}_{vi}(t)\widetilde{p}_{ij}^{(n)}\widehat{p}_{jy}\widetilde{\xi}_{yz}(t)\sum_{e\in\mathcal{E}}\frac{b(z,e)b(v,e)}{k_z} - \sum_{i,j,v,y,z\in\mathcal{N}} \pi_v \widetilde{\xi}_{vi}(t)\widetilde{p}_{ij}^{(n)}\widetilde{p}_{jy}\widetilde{\xi}_{yz}(t)\sum_{e\in\mathcal{E}}\frac{b(z,e)b(v,e)}{k_z} \\
=& \sum_{i,j,v,y,z\in\mathcal{N},y\neq j} \pi_v \widetilde{\xi}_{vi}(t)\widetilde{p}_{ij}^{(n)}\sum_{\alpha\in\mathcal{E}}\frac{b(j,\alpha)b(y,\alpha)}{k_j g_\alpha}\widetilde{\xi}_{yz}(t)\sum_{e\in\mathcal{E}}\frac{b(z,e)b(v,e)}{k_z} \\
&+ \sum_{i,j,v,z\in\mathcal{N}} \pi_v \widetilde{\xi}_{vi}(t)\widetilde{p}_{ij}^{(n)}\sum_{\alpha\in\mathcal{E}}\frac{b(j,\alpha)}{k_j g_\alpha}\widetilde{\xi}_{jz}(t)\sum_{e\in\mathcal{E}}\frac{b(z,e)b(v,e)}{k_z} \\
&- \sum_{i,j,v,y,z\in\mathcal{N},y\neq j} \pi_v \widetilde{\xi}_{vi}(t)\widetilde{p}_{ij}^{(n)}\sum_{\alpha\in\mathcal{E}}\frac{b(j,\alpha)b(y,\alpha)}{k_j (g_\alpha-1)}\widetilde{\xi}_{yz}(t)\sum_{e\in\mathcal{E}}\frac{b(z,e)b(v,e)}{k_z} \\
\approx& -\frac{1}{\langle g\rangle}\sum_{i,j,v,y,z\in\mathcal{N},y\neq j} \pi_v \widetilde{\xi}_{vi}(t)\widetilde{p}_{ij}^{(n)}\sum_{e\in\mathcal{E}}\frac{b(j,\alpha)b(y,\alpha)}{k_j (g_\alpha-1)}\widetilde{\xi}_{yz}(t)\sum_{e\in\mathcal{E}}\frac{b(z,e)b(v,e)}{k_z} \\
&+ \frac{1}{\langle g\rangle}\sum_{i,j,v,z\in\mathcal{N}} \pi_v \widetilde{\xi}_{vi}(t)\widetilde{p}_{ij}^{(n)}\sum_{\alpha\in\mathcal{E}}\frac{b(j,\alpha)}{k_j}\widetilde{\xi}_{jz}(t)\sum_{e\in\mathcal{E}}\frac{b(z,e)b(v,e)}{k_z} \\
=& -\frac{1}{\langle g\rangle}\sum_{i,j,v\in\mathcal{N}} \pi_v \widetilde{\xi}_{vi}(2t)\widetilde{p}_{ij}^{(n+1)}\sum_{e\in\mathcal{E}}\frac{b(j,e)b(v,e)}{k_j} + \frac{1}{\langle g\rangle}\sum_{i,j,v\in\mathcal{N}} \pi_v \widetilde{\xi}_{vi}(2t)\widetilde{p}_{ij}^{(n)}\sum_{e\in\mathcal{E}}\frac{b(j,e)b(v,e)}{k_j} \\
=& -\frac{1}{\langle g\rangle}\widetilde{A}_r^{(0,0,n+1,0)} + \frac{1}{\langle g\rangle}\widetilde{A}_r^{(0,0,n,0)}
\end{aligned} \tag{S36}$$

$$\begin{aligned}
\widetilde{A}_r^{(0,0,n,1)} - \widetilde{A}_r^{(0,0,n+1,0)} =& \sum_{i,j,v,y,z\in\mathcal{N}} \pi_v \widetilde{\xi}_{vi}(t)\widetilde{\xi}_{ij}(t)\widetilde{p}_{jy}^{(n)}\widehat{p}_{yz}\sum_{e\in\mathcal{E}}\frac{b(z,e)b(v,e)}{k_z} - \sum_{i,j,v,y,z\in\mathcal{N}} \pi_v \widetilde{\xi}_{vi}(t)\widetilde{\xi}_{ij}(t)\widetilde{p}_{jy}^{(n)}\widetilde{p}_{yz}\sum_{e\in\mathcal{E}}\frac{b(z,e)b(v,e)}{k_z} \\
=& \sum_{i,j,v,y,z\in\mathcal{N},z\neq y} \pi_v \widetilde{\xi}_{vi}(t)\widetilde{\xi}_{ij}(t)\widetilde{p}_{jy}^{(n)}\sum_{\alpha\in\mathcal{E}}\frac{b(y,\alpha)b(z,\alpha)}{k_y g_\alpha}\sum_{e\in\mathcal{E}}\frac{b(z,e)b(v,e)}{k_z} \\
&+ \sum_{i,j,v,y\in\mathcal{N}} \pi_v \widetilde{\xi}_{vi}(t)\widetilde{\xi}_{ij}(t)\widetilde{p}_{jy}^{(n)}\sum_{\alpha\in\mathcal{E}}\frac{b(y,\alpha)}{k_y g_\alpha}\sum_{e\in\mathcal{E}}\frac{b(y,e)b(v,e)}{k_z} \\
&- \sum_{i,j,v,y,z\in\mathcal{N},z\neq y} \pi_v \widetilde{\xi}_{vi}(t)\widetilde{\xi}_{ij}(t)\widetilde{p}_{jy}^{(n)}\sum_{\alpha\in\mathcal{E}}\frac{b(y,\alpha)b(z,\alpha)}{k_y (g_\alpha-1)}\sum_{e\in\mathcal{E}}\frac{b(z,e)b(v,e)}{k_z} \\
\approx& -\frac{1}{\langle g\rangle}\sum_{i,j,v,y,z\in\mathcal{N},z\neq y} \pi_v \widetilde{\xi}_{vi}(t)\widetilde{\xi}_{ij}(t)\widetilde{p}_{jy}^{(n)}\sum_{\alpha\in\mathcal{E}}\frac{b(y,\alpha)b(z,\alpha)}{k_y (g_\alpha-1)}\sum_{e\in\mathcal{E}}\frac{b(z,e)b(v,e)}{k_z} \\
&+ \frac{1}{\langle g\rangle} + \sum_{i,j,v,y\in\mathcal{N}} \pi_v \widetilde{\xi}_{vi}(t)\widetilde{\xi}_{ij}(t)\widetilde{p}_{jy}^{(n)}\sum_{\alpha\in\mathcal{E}}\frac{b(y,\alpha)}{k_y}\sum_{e\in\mathcal{E}}\frac{b(y,e)b(v,e)}{k_z} \\
=& -\frac{1}{\langle g\rangle}\sum_{i,j,v\in\mathcal{N}} \pi_v \widetilde{\xi}_{vi}(2t)\widetilde{p}_{ij}^{(n+1)}\sum_{e\in\mathcal{E}}\frac{b(j,e)b(v,e)}{k_j} + \frac{1}{\langle g\rangle}\sum_{i,j,v\in\mathcal{N}} \pi_v \widetilde{\xi}_{vi}(2t)\widetilde{p}_{ij}^{(n)}\sum_{e\in\mathcal{E}}\frac{b(j,e)b(v,e)}{k_j} \\
=& -\frac{1}{\langle g\rangle}\widetilde{A}_r^{(0,0,n+1,0)} + \frac{1}{\langle g\rangle}\widetilde{A}_r^{(0,0,n,0)}
\end{aligned} \tag{S37}$$

Thus, it is simple to yield that $\widetilde{A}_r^{(n,1,0,0)} \approx \widetilde{A}_r^{(0,0,n,1)}$. Given the above two steps of approximations, we have $\widetilde{A}_r^{(n,1,0,0)} \approx \widetilde{A}_r^{(0,0,n,1)}$. Subsequently, the term on the right of Eq. [S31] can be simplified as

$$\int_0^\infty \sum_{i,j,v,y\in\mathcal{N}} \pi_v \widetilde{\xi}_{vi}(t)p_{ij}^{(n,1)}\widetilde{\xi}_{jy}(t)\sum_{e\in\mathcal{E}}\frac{b(y,e)b(v,e)}{k_y}dt \approx \frac{1}{2}\int_0^\infty \sum_{i,j,v\in\mathcal{N}} \pi_v \widetilde{\xi}_{vi}(t)p_{ij}^{(n,1)}\sum_{e\in\mathcal{E}}\frac{b(j,e)b(v,e)}{k_y}dt \tag{S38}$$

Substituting the approximations of [S30] and [S38] into the condition for cooperation [S24], and then discretizing the time

series in units of one yield the simplified condition as

$$\begin{aligned}&\Big(\sum_{t=0}^{\infty}\sum_{i,j,v\in\mathcal{N}}\pi_v\widetilde{\xi}_{vi}^{(t)}p_{ij}^{(0,1)}\sum_{e\in\mathcal{E}}\frac{b(j,e)b(v,e)}{k_j}-\sum_{t=0}^{\infty}\sum_{i,j,v\in\mathcal{N}}\pi_v\widetilde{\xi}_{vi}^{(t)}p_{ij}^{(1,1)}\sum_{e\in\mathcal{E}}\frac{b(j,e)b(v,e)}{k_j}\Big)\cdot r\\&>\sum_{t=0}^{\infty}\sum_{i,v\in\mathcal{N}}\pi_v\widetilde{\xi}_{vi}^{(t)}p_{iv}^{(0,1)}-\sum_{t=0}^{\infty}\sum_{i,v\in\mathcal{N}}\pi_v\widetilde{\xi}_{vi}^{(t)}p_{iv}^{(1,1)}\end{aligned}\tag{S39}$$

5.2 Eliminating infinite summation

According to the form of [S39], we will eliminate infinite summation by dislocation subtraction. We begin with the second term on the left of the inequality [S39] as

$$\begin{aligned}\sum_{t=0}^{\infty}\sum_{i,j,v\in\mathcal{N}}\pi_v\widetilde{\xi}_{vi}^{(t)}p_{ij}^{(1,1)}\sum_{e\in\mathcal{E}}\frac{b(j,e)b(v,e)}{k_j}&=\sum_{t=0}^{\infty}\sum_{i,j,v,y\in\mathcal{N}}\pi_v\left(\widetilde{\Xi}^t\right)_{vi}\left(\widetilde{P}\right)_{iy}\widehat{p}_{yj}\sum_{e\in\mathcal{E}}\frac{b(j,e)b(v,e)}{k_j}\\&=\sum_{t=0}^{\infty}\sum_{i,j,v,y\in\mathcal{N}}\pi_v\left(\widetilde{\Xi}^t\right)_{vi}\left(N\widetilde{\Xi}-(N-1)E\right)_{iy}\widehat{p}_{yj}\sum_{e\in\mathcal{E}}\frac{b(j,e)b(v,e)}{k_j}.\\&=\sum_{t=0}^{\infty}\sum_{j,v,y\in\mathcal{N}}\pi_v\left[N\widetilde{\xi}_{vy}^{(t+1)}-(N-1)\widetilde{\xi}_{vy}^{(t)}\right]\widehat{p}_{yj}\sum_{e\in\mathcal{E}}\frac{b(j,e)b(v,e)}{k_j}\end{aligned}\tag{S40}$$

In order to make the term of [S40] converge to 0 as time goes to infinity, we subtract the formula $\lim_{t\to\infty}\widetilde{\xi}_{vy}^{(t)}=\pi_y$ as

$$\begin{aligned}&\sum_{t=0}^{\infty}\sum_{j,v,y\in\mathcal{N}}\pi_v\left[N\widetilde{\xi}_{vy}^{(t+1)}-(N-1)\widetilde{\xi}_{vy}^{(t)}\right]\widehat{p}_{yj}\sum_{e\in\mathcal{E}}\frac{b(j,e)b(v,e)}{k_j}-\sum_{t=0}^{\infty}\sum_{j,v,y\in\mathcal{N}}\pi_v\pi_y\widehat{p}_{yj}\sum_{e\in\mathcal{E}}\frac{b(j,e)b(v,e)}{k_j}\\&=\sum_{t=0}^{\infty}\sum_{j,u,y\in\mathcal{N}}\pi_u\left[N\left(\widetilde{\xi}_{uy}^{(t+1)}-\pi_y\right)-(N-1)\left(\widetilde{\xi}_{uy}^{(t)}-\pi_y\right)\right]\widehat{p}_{yj}\sum_{e\in\mathcal{E}}\frac{b(j,e)b(v,e)}{k_j}\\&=N\sum_{t=1}^{\infty}\sum_{j,u,y\in\mathcal{N}}\pi_u\left(\widetilde{\xi}_{uy}^{(t)}-\pi_y\right)\widehat{p}_{yj}\sum_{e\in\mathcal{E}}\frac{b(j,e)b(v,e)}{k_j}-(N-1)\sum_{t=0}^{\infty}\sum_{j,u,y\in\mathcal{N}}\pi_u\left(\widetilde{\xi}_{uy}^{(t)}-\pi_y\right)\widehat{p}_{yj}\sum_{e\in\mathcal{E}}\frac{b(j,e)b(v,e)}{k_j}\end{aligned}.\tag{S41}$$

Analogously, we subtract $\lim_{t\to\infty}\widetilde{\xi}_{vi}^{(t)}=\pi_i$ from the first term on the left of the inequality [S39], thus yielding

$$\begin{aligned}&\sum_{t=0}^{\infty}\sum_{i,j,v\in\mathcal{N}}\pi_v\widetilde{\xi}_{vi}^{(t)}p_{ij}^{(0,1)}\sum_{e\in\mathcal{E}}\frac{b(j,e)b(v,e)}{k_j}-\sum_{t=0}^{\infty}\sum_{i,j,v\in\mathcal{N}}\pi_v\pi_i\widehat{p}_{ij}\sum_{e\in\mathcal{E}}\frac{b(j,e)b(v,e)}{k_j}\\&=N\sum_{t=0}^{\infty}\sum_{i,j,v\in\mathcal{N}}\pi_v\left(\widetilde{\xi}_{vi}^{(t)}-\pi_i\right)\widehat{p}_{ij}\sum_{e\in\mathcal{E}}\frac{b(j,e)b(v,e)}{k_j}-(N-1)\sum_{t=0}^{\infty}\sum_{i,j,v\in\mathcal{N}}\pi_v\left(\widetilde{\xi}_{vi}^{(t)}-\pi_i\right)\widehat{p}_{ij}\sum_{e\in\mathcal{E}}\frac{b(j,e)b(v,e)}{k_j}\end{aligned}.\tag{S42}$$

Combining [S41] with [S42], it is easy to recast the left terms of the inequality [S39] into the following form as

$$\begin{aligned}
&\sum_{t=0}^{\infty}\sum_{i,j,v\in\mathcal{N}}\pi_v\widetilde{\xi}_{vi}^{(t)}p_{ij}^{(0,1)}\sum_{e\in\mathcal{E}}\frac{b(j,e)b(v,e)}{k_j}-\sum_{t=0}^{\infty}\sum_{i,j,v\in\mathcal{N}}\pi_v\widetilde{\xi}_{vi}^{(t)}p_{ij}^{(1,1)}\sum_{e\in\mathcal{E}}\frac{b(j,e)b(v,e)}{k_j}\\
=&\left(\sum_{t=0}^{\infty}\sum_{i,j,v\in\mathcal{N}}\pi_v\widetilde{\xi}_{vi}^{(t)}p_{ij}^{(0,1)}\sum_{e\in\mathcal{E}}\frac{b(j,e)b(v,e)}{k_j}-\sum_{t=0}^{\infty}\sum_{i,j,v\in\mathcal{N}}\pi_v\pi_i\widehat{p}_{ij}\sum_{e\in\mathcal{E}}\frac{b(j,e)b(v,e)}{k_j}\right)\\
&-\left(\sum_{t=0}^{\infty}\sum_{i,j,v\in\mathcal{N}}\pi_v\widetilde{\xi}_{vi}^{(t)}p_{ij}^{(1,1)}\sum_{e\in\mathcal{E}}\frac{b(j,e)b(v,e)}{k_j}-\sum_{t=0}^{\infty}\sum_{i,j,v\in\mathcal{N}}\pi_v\pi_i\widehat{p}_{ij}\sum_{e\in\mathcal{E}}\frac{b(j,e)b(v,e)}{k_j}\right)\\
=&N\left(\sum_{i,v\in\mathcal{N}}\pi_v p_{vi}^{(0,1)}\sum_{e\in\mathcal{E}}\frac{b(i,e)b(v,e)}{k_i}-\sum_{i,v\in\mathcal{N}}\pi_v\pi_i\sum_{e\in\mathcal{E}}\frac{b(i,e)b(v,e)}{k_i}\right)
\end{aligned}. \tag{S43}$$

As for the right terms of the inequality [S39], we adopt the similar manner of subtracting $\lim_{t\to\infty}\widetilde{\xi}_{vi}^{(t)}=\pi_i$ from these terms such that they can converge to 0 as time goes to infinity. Hence, the right terms of the inequality [S39] can be recast into

$$\sum_{t=0}^{\infty}\sum_{i,v\in\mathcal{N}}\pi_v\widetilde{\xi}_{vi}^{(t)}p_{iv}^{(0,1)}-\sum_{t=0}^{\infty}\sum_{i,v\in\mathcal{N}}\pi_v\widetilde{\xi}_{vi}^{(t)}p_{iv}^{(1,1)}=N\left(\sum_{i,v\in\mathcal{N}}\pi_v p_{vv}^{(0,1)}-\sum_{v\in\mathcal{N}}\pi_v^2\right) \tag{S44}$$

Based on the above transformations, we have the condition of cooperation under GMC mechanism with limited terms as

$$\left(\sum_{i,v\in\mathcal{N}}\pi_v p_{vi}^{(0,1)}\sum_{e\in\mathcal{E}}\frac{b(i,e)b(v,e)}{k_i}-\sum_{i,v\in\mathcal{N}}\pi_v\pi_i\sum_{e\in\mathcal{E}}\frac{b(i,e)b(v,e)}{k_i}\right)\cdot r>\sum_{i,v\in\mathcal{N}}\pi_v p_{vv}^{(0,1)}-\sum_{v\in\mathcal{N}}\pi_v^2. \tag{S45}$$

5.3 Critical synergy factor

According to the condition [S45], we obtain the critical synergy factor required for cooperation under GMC mechanism as

$$r^*=\frac{\sum_{v\in\mathcal{N}}\pi_v p_{vv}^{(0,1)}-\sum_{v\in\mathcal{N}}\pi_v^2}{\sum_{i,v\in\mathcal{N}}\pi_v p_{vi}^{(0,1)}\sum_{e\in\mathcal{E}}\frac{b(i,e)b(v,e)}{k_i}-\sum_{i,v\in\mathcal{N}}\pi_v\pi_i\sum_{e\in\mathcal{E}}\frac{b(i,e)b(v,e)}{k_i}}. \tag{S46}$$

When the positive synergy factor exceeds r^*, cooperation is dominant over defection under GMC mechanism as the trait evolves. We further simplify the critical threshold of GMC such that the theoretical result is directly related to the network parameters. Let $\langle x\rangle$ and $\langle x^2\rangle$ denote the first moment of x and the second moment of x, respectively. Each term in the critical synergy factor [S46] can be transformed into

$$\sum_{v\in\mathcal{N}}\pi_v^2=\frac{\langle k^2\rangle}{N\langle k\rangle^2}, \tag{S47}$$

$$\sum_{v\in\mathcal{N}}\pi_v p_{vv}^{(0,1)}=\sum_{v\in\mathcal{N}}\pi_v\sum_{e\in\mathcal{E}}\frac{b(v,e)b(v,e)}{k_v g_e}=\sum_{v\in\mathcal{N}}\frac{\pi_v}{k_v}\sum_{e\in\mathcal{E}}\frac{b(e,v)}{g_e}=\frac{1}{\sum_{i\in\mathcal{N}}k_i}\sum_{e\in\mathcal{E}}\frac{\sum_{v\in\mathcal{N}}b(v,e)}{g_e}=\frac{\sum_{e\in\mathcal{E}}1}{\sum_{e\in\mathcal{E}}g_e}=\frac{1}{\langle g\rangle}, \tag{S48}$$

$$\sum_{i,v\in\mathcal{N}}\pi_v p_{vi}^{(0,1)}\sum_{e\in\mathcal{E}}\frac{b(i,e)b(v,e)}{k_i}=\frac{1}{\langle k\rangle}+\frac{1}{N\langle k\rangle}\sum_{\alpha,e\in\mathcal{E},\alpha\neq e}\sum_{i,v\in\mathcal{N}}\frac{b(v,\alpha)b(i,\alpha)b(i,e)b(v,e)}{k_i g_\alpha}, \tag{S49}$$

$$\sum_{i,v\in\mathcal{N}}\pi_v\pi_i\sum_{e\in\mathcal{E}}\frac{b(i,e)b(v,e)}{k_i}=\frac{1}{N^2\langle k\rangle^2}\sum_{e\in\mathcal{E}}\sum_{v\in\mathcal{N}}k_v b(v,e)g_e. \tag{S50}$$

where $\langle k\rangle=\sum_{i\in\mathcal{N}}k_i/N$ represents the average hyperdegree of the hypergraph; $\langle k^2\rangle=\sum_{i\in\mathcal{N}}k_i^2/N$ represents the second moment of the hyperdegree of the hypergraph; and $\langle g\rangle=\sum_{e\in\mathcal{E}}g_e/L$ represents the average order of the hypergraph.

Next, we define the hyperdegree heterogeneity η_k, the assortativity coefficient ζ, and the overlap between hyperedges $\widehat{\theta}$ respectively as

$$\eta_k=\frac{\langle k^2\rangle}{\langle k\rangle^2}, \tag{S51}$$

$$\zeta=\frac{1}{N\langle k\rangle^2}\sum_{e\in\mathcal{E}}\sum_{v\in\mathcal{N}}k_v b(v,e)g_e=\frac{1}{N\langle k\rangle^2}\sum_{e\in\mathcal{E}}\sum_{v\in e}k_v g_e, \tag{S52}$$

$$\widehat{\theta}=\frac{1}{N\langle k\rangle}\sum_{\alpha,e\in\mathcal{E},\alpha\neq e}\sum_{i,v\in\mathcal{N}}\frac{b(v,\alpha)b(i,\alpha)b(i,e)b(v,e)}{k_i g_\alpha}=\frac{1}{N\langle k\rangle}\sum_{\alpha,e\in\mathcal{E},\alpha\neq e}\sum_{i\in\alpha\cap\beta}\frac{|\alpha\cap e|}{k_i g_\alpha}, \tag{S53}$$

where $|\alpha\cap e|$ represents the number of nodes in the intersection of hyperedges α and e. For the parameter $\widehat{\theta}$,

$$\frac{1}{g}\left(1-\frac{1}{\langle k\rangle}\right)<\widehat{\theta}<1-\frac{1}{\langle k\rangle}.$$

Hence, the critical synergy factor required to support cooperation under GMC mechanism can be rewritten as

$$r^*=\frac{\dfrac{N}{\langle g\rangle}-\eta_k}{\dfrac{N}{\langle k\rangle}+N\widehat{\theta}-\zeta}. \tag{S54}$$

6 Other higher-order update mechanisms

6.1 Higher-order death-birth mechanism

6.1.1 Fixation probability under weak selection

The higher-order death-birth (HDB) mechanism describes group-and-individual-biased selection of a to-be-updated node's neighbor to imitate. Under HDB mechanism and the state $\mathbf{s}$, the to-be-updated node i firstly selects a hyperedge e it belongs to with the probability

$$\Pr[i\to e](\mathbf{s})=\frac{b(i,e)F_{e\setminus\{i\}}(\mathbf{s})}{\sum_{\alpha\in\mathcal{E}}b(i,\alpha)F_{\alpha\setminus\{i\}}(\mathbf{s})}, \tag{S55}$$

where $F_{e\setminus\{i\}}=\sum_{j\in\mathcal{N},j\neq i}\widetilde{q}(e,j)f_j(\mathbf{s})$ represents the average fitness of the members in hyperedge e except node i.

Subsequently, the node i selects a neighbor j $(j \neq i)$ in the hyperedge e with the probability

$$\Pr\left[i \to j | i \to e\right](\mathbf{s}) = \frac{b(j,e)f_j(\mathbf{s})}{\sum_{y\in\mathcal{N}, y\neq i} b(y,e)f_y(\mathbf{s})}. \tag{S56}$$

Finally, the node i updates one's strategy by imitating the node j, thus following the probability

$$\begin{aligned}
\Pr\left[i \to j\right](\mathbf{s}) &= \sum_{e\in\mathcal{E}} \Pr\left[i \to e\right](\mathbf{s}) \cdot \Pr\left[i \to j | i \to e\right](\mathbf{s}) \\
&= \sum_{e\in\mathcal{E}} \frac{b(i,e)F_{e\setminus\{i\}}(\mathbf{s})}{\sum_{\alpha\in\mathcal{E}} b(i,\alpha)F_{\alpha\setminus\{i\}}(\mathbf{s})} \cdot \frac{b(j,e)f_j(\mathbf{s})}{\sum_{y\in\mathcal{N}, y\neq i} b(y,e)f_y(\mathbf{s})} \\
&= \sum_{e\in\mathcal{E}} \frac{\frac{b(i,e)}{k_i}\sum_{y\in\mathcal{N}, y\neq i} \widetilde{q}(e,y)f_y(\mathbf{s})}{\sum_{\alpha\in\mathcal{E}} \frac{b(i,\alpha)}{k_i}\sum_{y\in\mathcal{N}, y\neq i} \widetilde{q}(\alpha,y)f_y(\mathbf{s})} \cdot \frac{\frac{b(j,e)}{g_e-1}f_j(\mathbf{s})}{\sum_{y\in\mathcal{N}, y\neq i} \frac{b(y,e)}{g_e-1}f_y(\mathbf{s})} \\
&= \sum_{e\in\mathcal{E}} \frac{q(i,e)\sum_{y\in\mathcal{N}, y\neq i} \widetilde{q}(e,y)f_y(\mathbf{s})}{\sum_{\alpha\in\mathcal{E}} q(i,\alpha)\sum_{y\in\mathcal{N}, y\neq i} \widetilde{q}(\alpha,y)f_y(\mathbf{s})} \cdot \frac{\widetilde{q}(e,j)f_j(\mathbf{s})}{\sum_{y\in\mathcal{N}, y\neq i} \widetilde{q}(e,y)f_y(\mathbf{s})} \\
&= \sum_{e\in\mathcal{E}} \frac{q(i,e)\widetilde{q}(e,j)f_j(\mathbf{s})}{\sum_{\alpha\in\mathcal{E}} q(i,\alpha)\sum_{y\in\mathcal{N}, y\neq i} \widetilde{q}(\alpha,y)F_y(\mathbf{s})} \\
&= \frac{\widetilde{p}_{ij}f_j(\mathbf{s})}{\sum_{y\in\mathcal{N}} \widetilde{p}_{iy}f_y(\mathbf{s})}.
\end{aligned} \tag{S57}$$

Based on the transition probability [S57], the instantaneous change of the system's state is derived as

$$\begin{aligned}
\sum_{j\in\mathcal{N}} \pi_j \cdot \frac{ds_j^v(t)}{dt} &= \sum_{j\in\mathcal{N}} \pi_j \left(\sum_{i\in\mathcal{N}} \Pr[j \to i]\left(\mathbf{s}^v(t)\right) \cdot s_i^v(t) - s_j^v(t) \right) \\
&= \sum_{j\in\mathcal{N}} \pi_j \left(\sum_{i\in\mathcal{N}} s_i^v(t) \frac{\widetilde{p}_{ji}f_i(\mathbf{s}^v(t))}{\sum_{y\in\mathcal{N}} \widetilde{p}_{jy}f_y(\mathbf{s}^v(t))} - s_j^v(t) \right) \\
&= \sum_{i\in\mathcal{N}} s_i^v(t) \left(\sum_{j\in\mathcal{N}} \pi_j \frac{\widetilde{p}_{ji}f_i(\mathbf{s}^v(t))}{\sum_{y\in\mathcal{N}} \widetilde{p}_{jy}f_y(\mathbf{s}^v(t))} - \pi_i \right) \\
&= \delta \cdot \left[\sum_{i\in\mathcal{N}} \pi_i s_i^v(t) \left(u_i(\mathbf{s}^v(t)) - u_i(\mathbf{s}^v(t))^{(2,0)} \right) \right]_{\delta=0} + O(\delta^2).
\end{aligned} \tag{S58}$$

Substituting the instantaneous change of the system's state into the definition of fixation probability [S15] yields

$$\begin{aligned}
\rho_{\mathrm{C}} =& \frac{1}{N} + \frac{\delta}{N}\int_0^\infty \mathbb{E}\left[-\left(\sum_{i,v\in\mathcal{N}} \pi_i s_i^v(t) s_i^v(t)^{(0,0)} - \sum_{i,v\in\mathcal{N}} \pi_i s_i^v(t) s_i^v(t)^{(2,0)} \right) \right. \\
&\left. + \left(\sum_{i,j,y,v\in\mathcal{N}} \pi_i s_i^v(t) p_{ij}^{(0,0)} \sum_{e\in\mathcal{E}} \frac{b(j,e)b(y,e)}{k_j} s_y^v(t) - \sum_{i,j,y,v\in\mathcal{N}} \pi_i s_i^v(t) p_{ij}^{(2,0)} \sum_{e\in\mathcal{E}} \frac{b(j,e)b(y,e)}{k_j} s_y^v(t) \right) \cdot r \right]_{\delta=0} dt + O(\delta^2).
\end{aligned} \tag{S59}$$

6.1.2 Coalescence process in probabilistic sense

To approximate the probability of the node separately tracing the mutant ancestor, the node's state should be transformed into a probability of a node to be a cooperator. Thus, the condition that cooperation is dominant over defection (*i.e.*, $\rho_C > 1/N$) is given by

$$\begin{aligned} &\int_0^\infty \left[\sum_{i,j,y,v\in\mathcal{N}} \pi_i x_i^v(t) \left(p_{ij}^{(0,0)} \sum_{e\in\mathcal{E}} \frac{b(j,e)b(y,e)}{k_j} x_y^v(t) - p_{ij}^{(2,0)} \sum_{e\in\mathcal{E}} \frac{b(j,e)b(y,e)}{k_j} x_y^v(t) \right) \right]_{\delta=0} dt \cdot r \\ &> \int_0^\infty \left[\sum_{i,v\in\mathcal{N}} \pi_i x_i^v(t) \Big(x_i^v(t)^{(0,0)} - x_i^v(t)^{(2,0)} \Big) \right]_{\delta=0} dt \end{aligned} \tag{S60}$$

Under neutral drift ($\delta = 0$), the probability that the node i selects the neighbor j with HDB mechanism is

$$\big[\Pr[i \to j](\mathbf{x}^v(t))\big]_{\delta=0} = \widetilde{p}_{ij}. \tag{S61}$$

Therefore, the probabilistic state of the node can be traced back based on the transition matrix $\widetilde{\Xi}(t)$. For the node i, its probabilistic state at time t is derived as

$$\big[x_i^v(t)\big]_{\delta=0} = \sum_{j\in\mathcal{N}} \widetilde{\xi}_{ij}(t) x_j^v(0) = \widetilde{\xi}_{iv}(t) x_v^v(0) = \widetilde{\xi}_{iv}(t). \tag{S62}$$

After substituting [S62] into the condition [S60], we adopt the mean-field approach to validate $\widetilde{A}_l^{(n,0,0,0)} \approx \widetilde{A}_r^{(0,0,n,0)}$ in the left terms of [S60].

$$\begin{aligned} \widetilde{A}_l^{(n,0,0,0)} &= \sum_{i,j,v,y\in\mathcal{N}, j\neq y} \pi_v \widetilde{\xi}_{vi}(t) p_{ij}^{(n,0)} \sum_{e\in\mathcal{E}} \frac{b(j,e)b(e,y)}{k_j} \cdot \widetilde{\xi}_{yv}(t) \\ &= \sum_{i,j,v,y\in\mathcal{N}, j\neq y} \pi_v \widetilde{\xi}_{vi}(t) p_{ij}^{(n,0)} \sum_{e\in\mathcal{E}} \frac{b(j,e)b(e,y)}{k_j \cdot (g_e-1)} (g_e - 1) \cdot \widetilde{\xi}_{yv}(t) + \sum_{i,j,y\in\mathcal{N}} \pi_v \widetilde{\xi}_{vi}(t) p_{ij}^{(n,0)} \sum_{e\in\mathcal{E}} \frac{b(j,e)}{k_j} \widetilde{\xi}_{jv}(t) \\ &\approx \big(\langle g\rangle - 1\big) \sum_{i,j,y,v\in\mathcal{N}} \pi_v \widetilde{\xi}_{vi}(t) p_{ij}^{(n,0)} \widetilde{p}_{jy} \widetilde{\xi}_{yv}(t) + \sum_{i,j,v\in\mathcal{N}} \pi_v \widetilde{\xi}_{vi}(t) p_{ij}^{(n,0)} \widetilde{\xi}_{jv}(t) \\ &= \big(\langle g\rangle - 1\big) \sum_{i,v\in\mathcal{N}} \pi_v \widetilde{\xi}_{vi}(2t) p_{iv}^{(n+1,0)} + \sum_{i,j,v\in\mathcal{N}} \pi_v \widetilde{\xi}_{vi}(2t) p_{iv}^{(n,0)} \end{aligned} \tag{S63}$$

$$\begin{aligned} \widetilde{A}_r^{(0,0,n,0)} &= \sum_{i,j,v,y\in\mathcal{N}, y\neq v} \pi_v \widetilde{\xi}_{vi}(t) \widetilde{\xi}_{ij}(t) p_{jy}^{(n,0)} \sum_{e\in\mathcal{E}} \frac{b(y,e)b(v,e)}{k_y} \\ &= \sum_{i,j,v,y\in\mathcal{N}, y\neq v} \pi_v \widetilde{\xi}_{vi}(t) \widetilde{\xi}_{ij}(t) p_{jy}^{(n,0)} \sum_{e\in\mathcal{E}} \frac{b(y,e)b(v,e)}{k_y(g_e-1)} \cdot (g_e - 1) + \sum_{i,j,v\in\mathcal{N}} \pi_v \widetilde{\xi}_{vi}(t) \widetilde{\xi}_{ij}(t) p_{jv}^{(n,0)} \sum_{e\in\mathcal{E}} \frac{b(v,e)}{k_v} \\ &\approx \big(\langle g\rangle - 1\big) \sum_{i,j,y,v\in\mathcal{N}} \pi_v \widetilde{\xi}_{vi}(t) \widetilde{\xi}_{ij}(t) p_{jy}^{(n,0)} \widetilde{p}_{yv} + \sum_{i,j,v\in\mathcal{N}} \pi_v \widetilde{\xi}_{vi}(t) \widetilde{\xi}_{ij}(t) p_{jv}^{(n,0)} \\ &= \big(\langle g\rangle - 1\big) \sum_{i,v\in\mathcal{N}} \pi_v \widetilde{\xi}_{vi}(2t) p_{iv}^{(n+1,0)} + \sum_{i,j,v\in\mathcal{N}} \pi_v \widetilde{\xi}_{vi}(2t) p_{iv}^{(n,0)} \end{aligned} \tag{S64}$$

Thus, it is simple to yield that $\widetilde{A}_l^{(n,0,0,0)} \approx \widetilde{A}_r^{(0,0,n,0)}$. Under HDB mechanism, the condition for cooperation involves the

cases of $n = 0, 2$. Based on such approximations and also according to the derivation [S30], the condition for cooperation under HDB mechanism can be simplified as

$$\begin{aligned}&\left(\sum_{t=0}^{\infty}\sum_{i,j,v\in\mathcal{N}}\pi_v\widetilde{\xi}_{vi}^{(t)}p_{ij}^{(0,0)}\sum_{e\in\mathcal{E}}\frac{b(j,e)b(v,e)}{k_j}-\sum_{t=0}^{\infty}\sum_{i,j,v\in\mathcal{N}}\pi_v\widetilde{\xi}_{vi}^{(t)}p_{ij}^{(2,0)}\sum_{e\in\mathcal{E}}\frac{b(j,e)b(v,e)}{k_j}\right)\cdot r\\&>\sum_{t=0}^{\infty}\sum_{i,v\in\mathcal{N}}\pi_v\widetilde{\xi}_{vi}^{(t)}p_{iv}^{(0,0)}-\sum_{t=0}^{\infty}\sum_{i,v\in\mathcal{N}}\pi_v\widetilde{\xi}_{vi}^{(t)}p_{iv}^{(2,0)}\end{aligned}\tag{S65}$$

After dislocation elimination of infinite summations, the condition for cooperation under HDB mechanism is described as

$$\begin{aligned}&\left((-N^2+2N)\sum_{i,v\in\mathcal{N}}\pi_v\widetilde{\xi}_{vi}^{(0)}\sum_{e\in\mathcal{E}}\frac{b(i,e)b(v,e)}{k_i}+N^2\sum_{i,v\in\mathcal{N}}\pi_v\widetilde{\xi}_{vi}^{(1)}\sum_{e\in\mathcal{E}}\frac{b(i,e)b(v,e)}{k_i}-2N\sum_{i,v\in\mathcal{N}}\pi_v\pi_i\sum_{e\in\mathcal{E}}\frac{b(i,e)b(v,e)}{k_i}\right)\cdot r\\&>(-N^2+2N)\sum_{v\in\mathcal{N}}\pi_v\widetilde{\xi}_{vv}^{(0)}+N^2\sum_{v\in\mathcal{N}}\pi_v\widetilde{\xi}_{vv}^{(1)}-2N\sum_{v\in\mathcal{N}}\pi_v^2\end{aligned}\tag{S66}$$

6.1.3 Critical synergy factor

According to the relation [S26], the transition probabilities concerning with the elements of matrix $\widetilde{\Xi}$ can be replaced, and thus the critical synergy factor of HDB mechanism is obtained as

$$r^*=\frac{\sum_{v\in\mathcal{N}}\pi_v p_{vv}^{(0,0)}+\sum_{v\in\mathcal{N}}\pi_v p_{vv}^{(1,0)}-2\sum_{v\in\mathcal{N}}\pi_v^2}{\sum_{i,v\in\mathcal{N}}\pi_v p_{vi}^{(0,0)}\sum_{e\in\mathcal{E}}\dfrac{b(i,e)b(v,e)}{k_i}+\sum_{i,v\in\mathcal{N}}\pi_v p_{vi}^{(1,0)}\sum_{e\in\mathcal{E}}\dfrac{b(i,e)b(v,e)}{k_i}-2\sum_{i,v\in\mathcal{N}}\pi_v\pi_i\sum_{e\in\mathcal{E}}\dfrac{b(i,e)b(v,e)}{k_i}}.\tag{S67}$$

where cooperation is dominent over defection when the synergy factor exceeds the positive critical threshold.

For HDB mechanism, the critical synergy factor directly related to the hypergraph properties can be written as

$$r^*=\frac{N-2\eta_k}{N+\dfrac{N}{\langle k\rangle}+N\widetilde{\theta}-2\zeta}\tag{S68}$$

where the overlap $\widetilde{\theta}$ is defined as

$$\widetilde{\theta}=\frac{1}{N\langle k\rangle}\sum_{\alpha,e\in\mathcal{E},\alpha\neq e}\sum_{i,v\in\mathcal{N},i\neq v}\frac{b(v,\alpha)b(i,\alpha)b(i,e)b(v,e)}{k_i(g_\alpha-1)}=\frac{1}{N\langle k\rangle}\sum_{\alpha,e\in\mathcal{E},\alpha\neq e}\sum_{i\in\alpha\cap\beta}\frac{|\alpha\cap e|-1}{k_i(g_\alpha-1)}\tag{S69}$$

Above, $0<\widetilde{\theta}<1-\dfrac{1}{\langle k\rangle}$.

6.2 Higher-order imitation mechanism

6.2.1 Fixation probability under weak selection

The higher-order imitation (HIM) mechanism describes group-and-individual-biased selection of a target individual to imitate, where the to-be-updated node also can select oneself to keep its own strategy. Under HIM mechanism and the state $\mathbf{s}$, the

to-be-updated node i firstly selects a hyperedge e it belongs to with the probability

$$\Pr[i \to e](\mathbf{s}) = \frac{b(i,e)F_e(\mathbf{s})}{\sum_{\alpha\in\mathcal{E}} b(i,\alpha)F_\alpha(\mathbf{s})}, \tag{S70}$$

Subsequently, the node i selects a target individual j ($j = i$ is allowed) in the hyperedge e with the probability

$$\Pr\left[i \to j | i \to e\right](\mathbf{s}) = \frac{b(j,e)f_j(\mathbf{s})}{\sum_{y\in\mathcal{N}} b(y,e)f_y(\mathbf{s})}. \tag{S71}$$

Finally, the node i updates one's strategy by imitating the node j, thus following the probability

$$\begin{aligned}
\Pr[i \to j](\mathbf{s}) &= \sum_{e\in\mathcal{E}} \Pr[i \to e](\mathbf{s}) \cdot \Pr\left[i \to j | i \to e\right](\mathbf{s}) \\
&= \sum_{e\in\mathcal{E}} \frac{b(i,e)F_e(\mathbf{s})}{\sum_{\alpha\in\mathcal{E}} b(i,\alpha)F_\alpha(\mathbf{s})} \cdot \frac{b(j,e)f_j(\mathbf{s})}{\sum_{y\in\mathcal{N}} b(y,e)f_y(\mathbf{s})} \\
&= \sum_{e\in\mathcal{E}} \frac{\frac{b(i,e)}{k_i}\sum_{y\in\mathcal{N}} \widehat{q}(e,y)f_y(\mathbf{s})}{\sum_{\alpha\in\mathcal{E}} \frac{b(i,\alpha)}{k_i}\sum_{y\in\mathcal{N}} \widehat{q}(\alpha,y)f_y(\mathbf{s})} \cdot \frac{\frac{b(j,e)}{g_e}f_j(\mathbf{s})}{\sum_{y\in\mathcal{N},y\neq i} \frac{b(y,e)}{g_e}f_y(\mathbf{s})} \\
&= \sum_{e\in\mathcal{E}} \frac{q(i,e)\sum_{y\in\mathcal{N}} \widehat{q}(e,y)f_y(\mathbf{s})}{\sum_{\alpha\in\mathcal{E}} q(i,\alpha)\sum_{y\in\mathcal{N}} \widehat{q}(\alpha,y)f_y(\mathbf{s})} \cdot \frac{\widehat{q}(e,j)f_j(\mathbf{s})}{\sum_{y\in\mathcal{N}} \widehat{q}(e,y)f_y(\mathbf{s})} \\
&= \sum_{e\in\mathcal{E}} \frac{q(i,e)\widehat{q}(e,j)f_j(\mathbf{s})}{\sum_{\alpha\in\mathcal{E}} q(i,\alpha)\sum_{y\in\mathcal{N}} \widehat{q}(\alpha,y)F_y(\mathbf{s})} \\
&= \frac{\widehat{p}_{ij}f_j(\mathbf{s})}{\sum_{y\in\mathcal{N}} \widehat{p}_{iy}f_y(\mathbf{s})}.
\end{aligned} \tag{S72}$$

Based on the transition probability [S72], the instantaneous change of the system's state is derived as

$$\begin{aligned}
\sum_{j\in\mathcal{N}} \pi_j \cdot \frac{ds_j^v(t)}{dt} &= \sum_{j\in\mathcal{N}} \pi_j \left(\sum_{i\in\mathcal{N}} \Pr[j \to i]\left(\mathbf{s}^v(t)\right) \cdot s_i^v(t) - s_j^v(t)\right) \\
&= \sum_{j\in\mathcal{N}} \pi_j \left(\sum_{i\in\mathcal{N}} s_i^v(t)\frac{\widehat{p}_{ji}f_i(\mathbf{s}^v(t))}{\sum_{y\in\mathcal{N}} \widehat{p}_{jy}f_y(\mathbf{s}^v(t))} - s_j^v(t)\right) \\
&= \sum_{i\in\mathcal{N}} s_i^v(t)\left(\sum_{j\in\mathcal{N}} \pi_j\frac{\widehat{p}_{ji}f_i(\mathbf{s}^v(t))}{\sum_{y\in\mathcal{N}} \widehat{p}_{jy}f_y(\mathbf{s}^v(t))} - \pi_i\right) \\
&= \delta \cdot \left[\sum_{i\in\mathcal{N}} \pi_i s_i^v(t)\left(u_i(\mathbf{s}^v(t)) - u_i(\mathbf{s}^v(t))^{(0,2)}\right)\right]_{\delta=0} + O(\delta^2).
\end{aligned} \tag{S73}$$

Substituting the instantaneous change of the system's state into the definition of fixation probability [S15] yields

$$
\begin{aligned}
\rho_{\mathrm{C}} =& \frac{1}{N} + \frac{\delta}{N}\int_0^{\infty} \mathbb{E}\left[-\Big(\sum_{i,v\in\mathcal{N}} \pi_i s_i^v(t) s_i^v(t)^{(0,0)} - \sum_{i,v\in\mathcal{N}} \pi_i s_i^v(t) s_i^v(t)^{(0,2)} \Big) \right. \\
&\left. + \Big(\sum_{i,j,y,v\in\mathcal{N}} \pi_i s_i^v(t) p_{ij}^{(0,0)} \sum_{e\in\mathcal{E}} \frac{b(j,e)b(y,e)}{k_j} s_y^v(t) - \sum_{i,j,y,v\in\mathcal{N}} \pi_i s_i^v(t) p_{ij}^{(0,2)} \sum_{e\in\mathcal{E}} \frac{b(j,e)b(y,e)}{k_j} s_y^v(t) \Big) \cdot r \right]_{\delta=0} dt + O(\delta^2).
\end{aligned} \tag{S74}
$$

6.2.2 Coalescence process in probabilistic sense

To approximate the probability of the node separately tracing the mutant ancestor, the node's state should be transformed into a probability of a node to be a cooperator. Thus, the condition that cooperation is dominant over defection (*i.e.*, $\rho_{\mathrm{C}} > 1/N$) is given by

$$
\begin{aligned}
&\int_0^{\infty} \left[\sum_{i,j,y,v\in\mathcal{N}} \pi_i x_i^v(t) \left(p_{ij}^{(0,0)} \sum_{e\in\mathcal{E}} \frac{b(j,e)b(y,e)}{k_j} x_y^v(t) - p_{ij}^{(0,2)} \sum_{e\in\mathcal{E}} \frac{b(j,e)b(y,e)}{k_j} x_y^v(t) \right) \right]_{\delta=0} dt \cdot r \\
&> \int_0^{\infty} \left[\sum_{i,v\in\mathcal{N}} \pi_i x_i^v(t) \Big(x_i^v(t)^{(0,0)} - x_i^v(t)^{(0,2)} \Big) \right]_{\delta=0} dt
\end{aligned} \tag{S75}
$$

Under neutral drift ($\delta = 0$), the probability that the node i selects the neighbor j with HIM mechanism is

$$
\left[\Pr[i \to j](\mathbf{x}^v(t)) \right]_{\delta=0} = \widehat{p}_{ij}. \tag{S76}
$$

Due to asynchronous update, the probability of state transition in a time step is

$$
\widehat{\xi}_{ij} = \frac{1}{N}\widehat{p}_{ij} + \left(1 + \frac{1}{N}\right) \tag{S77}
$$

The one-step transition matrix of HIM is

$$
\widehat{\Xi} = \frac{1}{N}\widehat{P} + \left(1 - \frac{1}{N}\right) E, \tag{S78}
$$

Introducing the time scale produces the matrix $\widehat{\Xi}(t)$, where each element represents the probability of the state transition during time t. For the node i, its probabilistic state at time t is derived as

$$
\left[x_i^v(t) \right]_{\delta=0} = \sum_{j\in\mathcal{N}} \widehat{\xi}_{ij}(t) x_j^v(0) = \widehat{\xi}_{iv}(t) x_v^v(0) = \widehat{\xi}_{iv}(t). \tag{S79}
$$

To solve the terms on the left of Eq. [S75], we introduce some notations for the following approximations.

$$
\widehat{A}_l^{(n_1,m_1,n_2,m_2)} = \sum_{i,j,y,z,v\in\mathcal{N}} \pi_v \widehat{\xi}_{vi}(t) p_{ij}^{(n_1,m_1)} \sum_{e\in\mathcal{E}} \frac{b(j,e)b(y,e)}{k_j} \widehat{\xi}_{yz}(t) p_{zv}^{(n_2,m_2)}; \tag{S80}
$$

$$
\widehat{A}_r^{(n_1,m_1,n_2,m_2)} = \sum_{i,j,y,z,v\in\mathcal{N}} \pi_v \widehat{\xi}_{vi}(t) p_{ij}^{(n_1,m_1)} \widehat{\xi}_{jy}(t) p_{yz}^{(n_2,m_2)} \sum_{e\in\mathcal{E}} \frac{b(z,e)b(v,e)}{k_z}. \tag{S81}
$$

The left term of Eq. [S75] to be solved can be represented as $\widehat{A}_l^{(0,m,0,0)}$. Then we adopt the mean-field approach to validate $\widehat{A}_l^{(0,m,0,0)} \approx \widehat{A}_r^{(0,0,0,m)}$ in the left terms of [S75].

$$\begin{aligned}
\widehat{A}_l^{(0,m,0,0)} &= \sum_{i,j,v,y\in\mathcal{N}} \pi_v \widehat{\xi}_{vi}(t) p_{ij}^{(0,m)} \sum_{e\in\mathcal{E}} \frac{b(j,e)b(e,y)}{k_j} \cdot \widehat{\xi}_{yv}(t) \\
&= \sum_{i,j,v,y\in\mathcal{N}} \pi_v \widehat{\xi}_{vi}(t) p_{ij}^{(0,m)} \sum_{e\in\mathcal{E}} \frac{b(j,e)b(e,y)}{k_j \cdot g_e} g_e \cdot \widehat{\xi}_{yv}(t) \\
&\approx \langle g \rangle \sum_{i,j,y,v\in\mathcal{N}} \pi_v \widehat{\xi}_{vi}(t) p_{ij}^{(0,m)} \widehat{p}_{jy} \widehat{\xi}_{yv}(t) \\
&= \langle g \rangle \sum_{i,v\in\mathcal{N}} \pi_v \widehat{\xi}_{vi}(2t) p_{iv}^{(0,n+1)}
\end{aligned} \tag{S82}$$

$$\begin{aligned}
\widehat{A}_r^{(0,0,0,m)} &= \sum_{i,j,v,y\in\mathcal{N}} \pi_v \widehat{\xi}_{vi}(t) \widehat{\xi}_{ij}(t) p_{jy}^{(0,n)} \sum_{e\in\mathcal{E}} \frac{b(y,e)b(v,e)}{k_y} \\
&= \sum_{i,j,v,y\in\mathcal{N}} \pi_v \widehat{\xi}_{vi}(t) \widehat{\xi}_{ij}(t) p_{jy}^{(0,n)} \sum_{e\in\mathcal{E}} \frac{b(y,e)b(v,e)}{k_y g_e} \cdot g_e \\
&\approx \langle g \rangle \sum_{i,j,v,y\in\mathcal{N}} \pi_v \widehat{\xi}_{vi}(t) \widehat{\xi}_{ij}(t) p_{jy}^{(0,n)} \widehat{p}_{yv} \\
&= \langle g \rangle \sum_{i,v\in\mathcal{N}} \pi_v \widehat{\xi}_{vi}(2t) p_{iv}^{(0,n+1)}
\end{aligned} \tag{S83}$$

Thus, it is simple to yield that $\widehat{A}_l^{(0,m,0,0)} \approx \widehat{A}_r^{(0,0,0,m)}$. Under HIM mechanism, the condition for cooperation involves the cases of $m = 0, 2$. Based on such approximations, the condition for cooperation under HIM mechanism can be simplified as

$$\begin{aligned}
&\left(\sum_{t=0}^{\infty} \sum_{i,j,v\in\mathcal{N}} \pi_v \widehat{\xi}_{vi}^{(t)} p_{ij}^{(0,0)} \sum_{e\in\mathcal{E}} \frac{b(j,e)b(v,e)}{k_j} - \sum_{t=0}^{\infty} \sum_{i,j,v\in\mathcal{N}} \pi_v \widehat{\xi}_{vi}^{(t)} p_{ij}^{(0,2)} \sum_{e\in\mathcal{E}} \frac{b(j,e)b(v,e)}{k_j} \right) \cdot r \\
&> \sum_{t=0}^{\infty} \sum_{i,v\in\mathcal{N}} \pi_v \widehat{\xi}_{vi}^{(t)} p_{iv}^{(0,0)} - \sum_{t=0}^{\infty} \sum_{i,v\in\mathcal{N}} \pi_v \widehat{\xi}_{vi}^{(t)} p_{iv}^{(0,2)}
\end{aligned} \tag{S84}$$

After dislocation elimination of infinite summations, the condition for cooperation under HIM mechanism is described as

$$\begin{aligned}
&\left((-N^2 + 2N) \sum_{i,v\in\mathcal{N}} \pi_v \widehat{\xi}_{vi}^{(0)} \sum_{e\in\mathcal{E}} \frac{b(i,e)b(v,e)}{k_i} + N^2 \sum_{i,v\in\mathcal{N}} \pi_v \widehat{\xi}_{vi}^{(1)} \sum_{e\in\mathcal{E}} \frac{b(i,e)b(v,e)}{k_i} - 2N \sum_{i,v\in\mathcal{N}} \pi_v \pi_i \sum_{e\in\mathcal{E}} \frac{b(i,e)b(v,e)}{k_i} \right) \cdot r \\
&> (-N^2 + 2N) \sum_{v\in\mathcal{N}} \pi_v \widehat{\xi}_{vv}^{(0)} + N^2 \sum_{v\in\mathcal{N}} \pi_v \widehat{\xi}_{vv}^{(1)} - 2N \sum_{v\in\mathcal{N}} \pi_v^2
\end{aligned} \tag{S85}$$

6.2.3 Critical synergy factor

According to the relation [S77], the transition probabilities concerning with the elemens of matrix $\widehat{\Xi}$ can be replaced, and thus the critical synergy factor of HIM mechanism is obtained as

$$r^* = \frac{\sum_{v\in\mathcal{N}} \pi_v p_{vv}^{(0,0)} + \sum_{v\in\mathcal{N}} \pi_v p_{vv}^{(0,1)} - 2\sum_{v\in\mathcal{N}} \pi_v^2}{\sum_{i,v\in\mathcal{N}} \pi_v p_{vi}^{(0,0)} \sum_{e\in\mathcal{E}} \frac{b(i,e)b(v,e)}{k_i} + \sum_{i,v\in\mathcal{N}} \pi_v p_{vi}^{(0,1)} \sum_{e\in\mathcal{E}} \frac{b(i,e)b(v,e)}{k_i} - 2\sum_{i,v\in\mathcal{N}} \pi_v \pi_i \sum_{e\in\mathcal{E}} \frac{b(i,e)b(v,e)}{k_i}}. \tag{S86}$$

where cooperation is dominent over defection when the synergy factor exceeds the positive critical threshold.

For HIM mechanism, the critical synergy factor directly related to the hypergraph properties can be written as

$$r^* = \frac{N + \dfrac{N}{\langle g \rangle} - 2\eta_k}{N + \dfrac{N}{\langle k \rangle} + N\widehat{\theta} - 2\zeta}. \tag{S87}$$

6.3 Group-inner comparison mechanism

6.3.1 Fixation probability under weak selection

The group-inner comparison (GIC) mechanism describes individual-biased selection of a target node that the to-be-updated node decides to imitate. Under GIC mechanism and the state $\mathbf{s}$, the to-be-updated node i firstly selects a hyperedge e it belongs to with the probability

$$\Pr[i \to e](\mathbf{s}) = \frac{b(i,e)}{k_i}, \tag{S88}$$

Subsequently, the node i selects a neighbor j ($j = i$ is allowed) in the hyperedge e with the probability

$$\Pr\left[i \to j | i \to e\right](\mathbf{s}) = \frac{b(j,e) f_j(\mathbf{s})}{\sum_{y \in \mathcal{N}} b(y,e) f_y(\mathbf{s})}. \tag{S89}$$

Finally, the node i updates one's strategy by imitating the node j, thus following the probability

$$\begin{aligned}
\Pr\left[i \to j\right](\mathbf{s}) &= \sum_{e \in \mathcal{E}} \Pr\left[i \to e\right](\mathbf{s}) \cdot \Pr\left[i \to j | i \to e\right](\mathbf{s}) \\
&= \sum_{e \in \mathcal{E}} \frac{b(i,e)}{k_i} \cdot \frac{\dfrac{b(j,e)}{g_e} f_j(\mathbf{s})}{\sum_{y \in \mathcal{N}} \dfrac{b(y,e)}{g_e} f_y(\mathbf{s})} \\
&= \sum_{e \in \mathcal{E}} q(i,e) \cdot \frac{\widehat{q}(e,j) f_j(\mathbf{s})}{\sum_{y \in \mathcal{N}} \widehat{q}(e,y) f_y(\mathbf{s})}.
\end{aligned} \tag{S90}$$

Based on the transition probability [S90], the instantaneous change of the system's state is derived as

$$\begin{aligned}
\sum_{j\in\mathcal{N}}\pi_j\cdot\frac{ds_j^v(t)}{dt} =& \sum_{j\in\mathcal{N}}\pi_j\bigg(\sum_{i\in\mathcal{N}}\Pr[j\to i]\left(\mathbf{s}^v(t)\right)\cdot s_i^v(t)-s_j^v(t)\bigg)\\
=&\sum_{j\in\mathcal{N}}\pi_j\bigg(\sum_{i\in\mathcal{N}}s_i^u(t)\sum_{e\in\mathcal{E}}q(j,e)\frac{\widehat{q}(e,i)f_i(\mathbf{s}^u(t))}{\sum_{x\in\mathcal{N}}\widehat{q}(e,x)f_x(\mathbf{s}^u(t))}-s_j^u(t)\bigg)\\
=&\delta\left[\sum_{i\in\mathcal{N}}s_i^u(t)\sum_{j\in\mathcal{N}}\pi_j\sum_{e\in\mathcal{E}}q(j,e)\widehat{q}(e,i)\bigg(u_i(\mathbf{s}^u(t))-\sum_{x\in\mathcal{N}}\widehat{q}(e,x)u_x(\mathbf{s}^u(t))\bigg)\right]_{\delta=0}+O(\delta^2)\\
=&\delta\left[\sum_{i,j\in\mathcal{N}}\pi_j\widehat{p}_{ji}s_i^u(t)f_i(\mathbf{s}^u(t))-\sum_{i\in\mathcal{N}}\sum_{e\in\mathcal{E}}s_i^u(t)\frac{k_i}{\sum_{y\in\mathcal{N}}k_y}\frac{\sum_{j\in\mathcal{N}}b(j,e)}{k_i}\frac{b(e,i)}{g_e}\frac{b(e,x)}{g_e}f_x(\mathbf{s}^u(t))\right]_{\delta=0}+O(\delta^2)\\
=&\delta\cdot\left[\sum_{i,j\in\mathcal{N}}s_i^u(t)\pi_j\widehat{p}_{ji}u_i(\mathbf{s}^u(t))-\sum_{i\in\mathcal{N}}\sum_{e\in\mathcal{E}}s_i^u(t)\pi_i\frac{b(i,e)}{k_i}\frac{b(e,x)}{g_e}u_x(\mathbf{s}^u(t))\right]_{\delta=0}+O(\delta^2)\\
=&\delta\cdot\left[\sum_{i\in\mathcal{V}}\pi_i s_i^u(t)\bigg(u_i(\mathbf{s}^u(t))-u_i(\mathbf{s}^u(t))^{(0,1)}\bigg)\right]_{\delta=0}+O(\delta^2).
\end{aligned}\tag{S91}$$

Substituting the instantaneous change of the system's state into the definition of fixation probability [S15] yields

$$\begin{aligned}
\rho_{\mathrm{C}}=&\frac{1}{N}+\frac{\delta}{N}\int_0^\infty\mathbb{E}\Bigg[-\bigg(\sum_{i,v\in\mathcal{N}}\pi_i s_i^v(t)s_i^v(t)^{(0,0)}-\sum_{i,v\in\mathcal{N}}\pi_i s_i^v(t)s_i^v(t)^{(0,1)}\bigg)\\
&+\bigg(\sum_{i,j,y,v\in\mathcal{N}}\pi_i s_i^v(t)p_{ij}^{(0,0)}\sum_{e\in\mathcal{E}}\frac{b(j,e)b(y,e)}{k_j}s_y^v(t)-\sum_{i,j,y,v\in\mathcal{N}}\pi_i s_i^v(t)p_{ij}^{(0,1)}\sum_{e\in\mathcal{E}}\frac{b(j,e)b(y,e)}{k_j}s_y^v(t)\bigg)\cdot r\Bigg]_{\delta=0}dt+O(\delta^2).
\end{aligned}\tag{S92}$$

6.3.2 Coalescence process in probabilistic sense

To approximate the probability of the node separately tracing the mutant ancestor, the node's state should be transformed into a probability of a node to be a cooperator. Thus, the condition that cooperation is dominant over defection (*i.e.*, $\rho_{\mathrm{C}}>1/N$) is given by

$$\begin{aligned}
&\int_0^\infty\left[\sum_{i,j,y,v\in\mathcal{N}}\pi_i x_i^v(t)\left(p_{ij}^{(0,0)}\sum_{e\in\mathcal{E}}\frac{b(j,e)b(y,e)}{k_j}x_y^v(t)-p_{ij}^{(0,1)}\sum_{e\in\mathcal{E}}\frac{b(j,e)b(y,e)}{k_j}x_y^v(t)\right)\right]_{\delta=0}dt\cdot r\\
&>\int_0^\infty\left[\sum_{i,v\in\mathcal{N}}\pi_i x_i^v(t)\bigg(x_i^v(t)^{(0,0)}-x_i^v(t)^{(0,1)}\bigg)\right]_{\delta=0}dt
\end{aligned}\tag{S93}$$

Under neutral drift ($\delta=0$), the probability that the node i selects the neighbor j with GIC mechanism is

$$\left[\Pr[i\to j](\mathbf{x}^v(t))\right]_{\delta=0}=\widehat{p}_{ij}.\tag{S94}$$

Therefore, the probabilistic state of the node can be traced back based on the transition matrix $\widehat{\Xi}(t)$. For the node i, its

probabilistic state at time t is derived as

$$\left[x_i^v(t)\right]_{\delta=0} = \sum_{j\in\mathcal{N}} \widehat{\xi}_{ij}(t)x_j^v(0) = \widehat{\xi}_{iv}(t)x_v^v(0) = \widehat{\xi}_{iv}(t). \tag{S95}$$

After substituting [S95] into the condition [S93], we adopt the mean-field approach to validate $\widehat{A}_l^{(0,m,0,0)} \approx \widehat{A}_r^{(0,0,0,m)}$ where $m = 0, 1$ in GIC mechanism. Based on such approximations, the condition for cooperation under GIC mechanism can be simplified as

$$\begin{aligned} &\left(\sum_{t=0}^{\infty}\sum_{i,j,v\in\mathcal{N}} \pi_v \widehat{\xi}_{vi}^{(t)} p_{ij}^{(0,0)} \sum_{e\in\mathcal{E}} \frac{b(j,e)b(v,e)}{k_j} - \sum_{t=0}^{\infty}\sum_{i,j,v\in\mathcal{N}} \pi_v \widehat{\xi}_{vi}^{(t)} p_{ij}^{(0,1)} \sum_{e\in\mathcal{E}} \frac{b(j,e)b(v,e)}{k_j}\right)\cdot r \\ &> \sum_{t=0}^{\infty}\sum_{i,v\in\mathcal{N}} \pi_v \widehat{\xi}_{vi}^{(t)} p_{iv}^{(0,0)} - \sum_{t=0}^{\infty}\sum_{i,v\in\mathcal{N}} \pi_v \widehat{\xi}_{vi}^{(t)} p_{iv}^{(0,1)} \end{aligned} \tag{S96}$$

After dislocation elimination of infinite summations, the condition for cooperation under GIC mechanism is described as

$$\left(\sum_{i,v\in\mathcal{N}} \pi_v p_{vi}^{(0,0)} \sum_{e\in\mathcal{E}} \frac{b(i,e)b(v,e)}{k_i} - \sum_{i,v\in\mathcal{N}} \pi_v \pi_i \sum_{e\in\mathcal{E}} \frac{b(i,e)b(v,e)}{k_i}\right)\cdot r > \sum_{i,v\in\mathcal{N}} \pi_v p_{vv}^{(0,0)} - \sum_{v\in\mathcal{N}} \pi_v^2. \tag{S97}$$

6.3.3 Critical synergy factor

According to the relation [S77], the transition probabilities concerning with the elements of matrix $\widehat{\Xi}$ can be replaced, and thus the critical synergy factor of GIC mechanism is obtained as

$$r^* = \frac{\sum_{v\in\mathcal{N}} \pi_v p_{vv}^{(0,0)} + \sum_{v\in\mathcal{N}} \pi_v p_{vv}^{(1,0)} - 2\sum_{v\in\mathcal{N}} \pi_v^2}{\sum_{i,v\in\mathcal{N}} \pi_v p_{vi}^{(0,0)} \sum_{e\in\mathcal{E}} \dfrac{b(i,e)b(v,e)}{k_i} - \sum_{i,v\in\mathcal{N}} \pi_v\pi_i \sum_{e\in\mathcal{E}} \dfrac{b(i,e)b(v,e)}{k_i}}. \tag{S98}$$

where cooperation is dominant over defection when the synergy factor exceeds the positive critical threshold.

For GIC mechanism, the critical synergy factor directly related to the hypergraph properties can be written as

$$r^* = \frac{N-\eta_k}{N-\zeta} \tag{S99}$$

6.4 Higher-order pair-comparison mechanism

6.4.1 Fixation probability under weak selection

The higher-order pair-comparison (HPC) mechanism describes non-biased selection of a to-be-updated node's neighbor to compare with. Under HPC mechanism and the state $\mathbf{s}$, the to-be-updated node i firstly selects a hyperedge e it belongs to with the probability

$$\Pr[i \to e](\mathbf{s}) = \frac{b(i,e)}{k_i}, \tag{S100}$$

Subsequently, the node i selects a target individual j $(j \neq i)$ in the hyperedge e with the probability

$$\Pr\left[i \to j | i \to e\right](\mathbf{s}) = \frac{b(j,e)}{g_e - 1}. \tag{S101}$$

Finally, the node i updates one's strategy by imitating the node j, thus following the probability

$$\begin{aligned}
\Pr\left[i \to j\right](\mathbf{s}) &= \sum_{e\in\mathcal{E}} \Pr\left[i \to e\right](\mathbf{s}) \cdot \Pr\left[i \to j | i \to e\right](\mathbf{s}) \\
&= \sum_{e\in\mathcal{E}} \frac{b(i,e)}{k_i} \cdot \frac{b(j,e)}{g_e - 1} \cdot \frac{f_j(\mathbf{s})}{f_i(\mathbf{s}) + f_j(\mathbf{s})} \\
&= \widetilde{p}_{ij} \cdot \frac{f_j(\mathbf{s})}{f_i(\mathbf{s}) + f_j(\mathbf{s})}.
\end{aligned} \tag{S102}$$

Based on the transition probability [S102], the instantaneous change of the system's state is derived as

$$\begin{aligned}
\sum_{j\in\mathcal{N}} \pi_j \cdot \frac{ds_j^v(t)}{dt} &= \sum_{j\in\mathcal{N}} \pi_j \left(\sum_{i\in\mathcal{N}} \Pr[j \to i] \left(\mathbf{s}^v(t)\right) \cdot s_i^v(t) - s_j^v(t) \right) \\
&= \sum_{j\in\mathcal{N}} \pi_j \left(\sum_{i\in\mathcal{N}} s_i^v(t) \widetilde{p}_{ji} \cdot \frac{f_i(\mathbf{s})}{f_i(\mathbf{s}) + f_j(\mathbf{s})} - s_j^v(t) \right) \\
&= \sum_{i\in\mathcal{N}} s_i^v(t) \left(\sum_{j\in\mathcal{N}} \pi_j \widetilde{p}_{ji} \cdot \frac{f_i(\mathbf{s})}{f_i(\mathbf{s}) + f_j(\mathbf{s})} - \pi_i \right) \\
&= \frac{\delta}{2} \cdot \left[\sum_{i\in\mathcal{N}} \pi_i s_i^v(t) \left(u_i(\mathbf{s}^v(t)) - u_i(\mathbf{s}^v(t))^{(1,0)} \right) \right]_{\delta=0} + O(\delta^2).
\end{aligned} \tag{S103}$$

Substituting the instantaneous change of the system's state into the definition of fixation probability [S15] yields

$$\begin{aligned}
\rho_{\mathrm{C}} =& \frac{1}{N} + \frac{\delta}{N} \int_0^\infty \mathbb{E} \left[-\left(\sum_{i,v\in\mathcal{N}} \pi_i s_i^v(t) s_i^v(t)^{(0,0)} - \sum_{i,v\in\mathcal{N}} \pi_i s_i^v(t) s_i^v(t)^{(1,0)} \right) \right. \\
&\left. + \left(\sum_{i,j,y,v\in\mathcal{N}} \pi_i s_i^v(t) p_{ij}^{(0,0)} \sum_{e\in\mathcal{E}} \frac{b(j,e)b(y,e)}{k_j} s_y^v(t) - \sum_{i,j,y,v\in\mathcal{N}} \pi_i s_i^v(t) p_{ij}^{(1,0)} \sum_{e\in\mathcal{E}} \frac{b(j,e)b(y,e)}{k_j} s_y^v(t) \right) \cdot r \right]_{\delta=0} dt + O(\delta^2).
\end{aligned} \tag{S104}$$

6.4.2 Coalescence process in probabilistic sense

To approximate the probability of the node separately tracing the mutant ancestor, the node's state should be transformed into a probability of a node to be a cooperator. Thus, the condition that cooperation is dominant over defection (*i.e.*, $\rho_{\mathrm{C}} > 1/N$) is given by

$$\begin{aligned}
&\int_0^\infty \left[\sum_{i,j,y,v\in\mathcal{N}} \pi_i x_i^v(t) \left(p_{ij}^{(0,0)} \sum_{e\in\mathcal{E}} \frac{b(j,e)b(y,e)}{k_j} x_y^v(t) - p_{ij}^{(1,0)} \sum_{e\in\mathcal{E}} \frac{b(j,e)b(y,e)}{k_j} x_y^v(t) \right) \right]_{\delta=0} dt \cdot r \\
&> \int_0^\infty \left[\sum_{i,v\in\mathcal{N}} \pi_i x_i^v(t) \left(x_i^v(t)^{(0,0)} - x_i^v(t)^{(1,0)} \right) \right]_{\delta=0} dt
\end{aligned} \tag{S105}$$

Under neutral drift ($\delta = 0$), the probability that the node i selects the neighbor j's strategy to update with HPC mechanism

is

$$\left[\Pr[i \to j](\mathbf{x}^v(t))\right]_{\delta=0} = \widetilde{p}_{ij}. \tag{S106}$$

Since the to-be-updated node can adopt its neighbor's strategy or keep one's own, the probability of state transition in a time step is

$$\mathring{\xi}_{ij} = \begin{cases} 1 - \dfrac{1}{2N} & i = j \\ \dfrac{1}{2N}\widetilde{p}_{ij} & i \neq j \end{cases}. \tag{S107}$$

The one-step transition matrix of HPC is

$$\mathring{\Xi} = \frac{1}{2N}\widetilde{P} + \left(1 - \frac{1}{2N}\right)E, \tag{S108}$$

Introducing the time scale produces the matrix $\mathring{\Xi}(t)$, where each element represents the probability of the state transition during time t. For the node i, its probabilistic state at time t is derived as

$$\left[x_i^v(t)\right]_{\delta=0} = \sum_{j\in\mathcal{N}} \mathring{\xi}_{ij}(t)x_j^v(0) = \mathring{\xi}_{iv}(t)x_v^v(0) = \mathring{\xi}_{iv}(t). \tag{S109}$$

To solve the terms on the left of the condition [S105], we introduce some notations for the following approximations.

$$\mathring{A}_l^{(n_1,m_1,n_2,m_2)} = \sum_{i,j,y,z,v\in\mathcal{N}} \pi_v \widetilde{\xi}_{vi}(t) p_{ij}^{(n_1,m_1)} \sum_{e\in\mathcal{E}} \frac{b(j,e)b(y,e)}{k_j} \widetilde{\xi}_{yz}(t) p_{zv}^{(n_2,m_2)}; \tag{S110}$$

$$\mathring{A}_r^{(n_1,m_1,n_2,m_2)} = \sum_{i,j,y,z,v\in\mathcal{N}} \pi_v \widetilde{\xi}_{vi}(t) p_{ij}^{(n_1,m_1)} \widetilde{\xi}_{jy}(t) p_{yz}^{(n_2,m_2)} \sum_{e\in\mathcal{E}} \frac{b(z,e)b(v,e)}{k_z}. \tag{S111}$$

The left term of the condition [S105] to be solved can be represented as $\mathring{A}_l^{(n,0,0,0)}$ where $n = 0, 1$ under HPC mechanism. Then we adopt the mean-field approach to validate $\mathring{A}_l^{(n,0,0,0)} \approx \mathring{A}_r^{(0,0,n,0)}$ in the left terms of [S105].

$$\begin{aligned}
\mathring{A}_l^{(n,0,0,0)} &= \sum_{i,j,v,y\in\mathcal{N}, j\neq y} \pi_v \mathring{\xi}_{vi}(t) p_{ij}^{(n,0)} \sum_{e\in\mathcal{E}} \frac{b(j,e)b(e,y)}{k_j} \cdot \mathring{\xi}_{yv}(t) \\
&= \sum_{i,j,v,y\in\mathcal{N}, j\neq y} \pi_v \mathring{\xi}_{vi}(t) p_{ij}^{(n,0)} \sum_{e\in\mathcal{E}} \frac{b(j,e)b(e,y)}{k_j \cdot (g_e - 1)} (g_e - 1) \cdot \mathring{\xi}_{yv}(t) + \sum_{i,j,y\in\mathcal{N}} \pi_v \mathring{\xi}_{vi}(t) p_{ij}^{(n,0)} \sum_{e\in\mathcal{E}} \frac{b(j,e)}{k_j} \mathring{\xi}_{jv}(t) \\
&\approx \left(\langle g\rangle - 1\right) \sum_{i,j,y,v\in\mathcal{N}} \pi_v \mathring{\xi}_{vi}(t) p_{ij}^{(n,0)} \mathring{p}_{jy} \mathring{\xi}_{yv}(t) + \sum_{i,j,v\in\mathcal{N}} \pi_v \mathring{\xi}_{vi}(t) p_{ij}^{(n,0)} \mathring{\xi}_{jv}(t) \\
&= \left(\langle g\rangle - 1\right) \sum_{i,v\in\mathcal{N}} \pi_v \mathring{\xi}_{vi}(2t) p_{iv}^{(n+1,0)} + \sum_{i,j,v\in\mathcal{N}} \pi_v \mathring{\xi}_{vi}(2t) p_{iv}^{(n,0)}
\end{aligned} \tag{S112}$$

$$\begin{aligned}
\mathring{A}_r^{(0,0,n,0)} &= \sum_{i,j,v,y\in\mathcal{N},y\neq v} \pi_v \mathring{\xi}_{vi}(t)\mathring{\xi}_{ij}(t)p_{jy}^{(n,0)} \sum_{e\in\mathcal{E}} \frac{b(y,e)b(v,e)}{k_y} \\
&= \sum_{i,j,v,y\in\mathcal{N},y\neq v} \pi_v \mathring{\xi}_{vi}(t)\mathring{\xi}_{ij}(t)p_{jy}^{(n,0)} \sum_{e\in\mathcal{E}} \frac{b(y,e)b(v,e)}{k_y(g_e-1)} \cdot (g_e-1) + \sum_{i,j,v\in\mathcal{N}} \pi_v \mathring{\xi}_{vi}(t)\mathring{\xi}_{ij}(t)p_{jv}^{(n,0)} \sum_{e\in\mathcal{E}} \frac{b(v,e)}{k_v} \\
&\approx \left(\langle g\rangle - 1\right) \sum_{i,j,y,v\in\mathcal{N}} \pi_v \mathring{\xi}_{vi}(t)\mathring{\xi}_{ij}(t)p_{jy}^{(n,0)}\mathring{p}_{yv} + \sum_{i,j,v\in\mathcal{N}} \pi_v \mathring{\xi}_{vi}(t)\mathring{\xi}_{ij}(t)p_{jv}^{(n,0)} \\
&= \left(\langle g\rangle - 1\right) \sum_{i,v\in\mathcal{N}} \pi_v \mathring{\xi}_{vi}(2t)p_{iv}^{(n+1,0)} + \sum_{i,j,v\in\mathcal{N}} \pi_v \mathring{\xi}_{vi}(2t)p_{iv}^{(n,0)}
\end{aligned} \tag{S113}$$

Thus, it is simple to yield that $\mathring{A}_l^{(n,0,0,0)} \approx \mathring{A}_r^{(0,0,n,0)}$. Under HPC mechanism, the condition for cooperation involves the cases of $n = 0, 1$. Based on such approximations, the condition for cooperation under HPC mechanism can be simplified as

$$\begin{aligned}
&\left(\sum_{t=0}^{\infty}\sum_{i,j,v\in\mathcal{N}} \pi_v \mathring{\xi}_{vi}^{(t)} p_{ij}^{(0,0)} \sum_{e\in\mathcal{E}} \frac{b(j,e)b(v,e)}{k_j} - \sum_{t=0}^{\infty}\sum_{i,j,v\in\mathcal{N}} \pi_v \mathring{\xi}_{vi}^{(t)} p_{ij}^{(1,0)} \sum_{e\in\mathcal{E}} \frac{b(j,e)b(v,e)}{k_j}\right) \cdot r \\
&> \sum_{t=0}^{\infty}\sum_{i,v\in\mathcal{N}} \pi_v \mathring{\xi}_{vi}^{(t)} p_{iv}^{(0,0)} - \sum_{t=0}^{\infty}\sum_{i,v\in\mathcal{N}} \pi_v \mathring{\xi}_{vi}^{(t)} p_{iv}^{(1,0)}
\end{aligned} \tag{S114}$$

After dislocation elimination of infinite summations, the condition for cooperation under GIC mechanism is described as

$$\left(\sum_{i,v\in\mathcal{N}} \pi_v p_{vi}^{(0,0)} \sum_{e\in\mathcal{E}} \frac{b(i,e)b(v,e)}{k_i} - \sum_{i,v\in\mathcal{N}} \pi_v\pi_i \sum_{e\in\mathcal{E}} \frac{b(i,e)b(v,e)}{k_i}\right) \cdot r > \sum_{i,v\in\mathcal{N}} \pi_v p_{vv}^{(0,0)} - \sum_{v\in\mathcal{N}} \pi_v^2. \tag{S115}$$

6.4.3 Critical synergy factor

According to the relation [S107], the transition probabilities concerning with the elements of matrix $\mathring{\Xi}$ can be replaced, and thus the critical synergy factor of HPC mechanism is obtained as

$$r^* = \frac{\sum_{v\in\mathcal{N}} \pi_v p_{vv}^{(0,0)} + \sum_{v\in\mathcal{N}} \pi_v p_{vv}^{(1,0)} - 2\sum_{v\in\mathcal{N}} \pi_v^2}{\sum_{i,v\in\mathcal{N}} \pi_v p_{vi}^{(0,0)} \sum_{e\in\mathcal{E}} \dfrac{b(i,e)b(v,e)}{k_i} - \sum_{i,v\in\mathcal{N}} \pi_v\pi_i \sum_{e\in\mathcal{E}} \dfrac{b(i,e)b(v,e)}{k_i}}. \tag{S116}$$

where cooperation is dominent over defection when the synergy factor exceeds the positive critical threshold.

For HPC mechanism, the critical synergy factor directly related to the hypergraph properties can be written as

$$r^* = \frac{N - \eta_k}{N - \zeta} \tag{S117}$$

7 Impact of hypergraph properties on evolutionary outcomes

In this section, we only consider the mechanisms of GMC, HDB and HIM, since GIC and HPC can certainly hinder cooperation.

7.1 Hyperdegree and order

For a homogeneous hypergraph, each node has the same hyperedgree and each hyperedge has the same hyperdegree. Hence, the hyperdegree heterogeneity $\eta_k = 1$, and the assortativity coefficient $\zeta = g$. And two forms of the overlap can be redefined as

$$\widetilde{\theta}_\circ = \frac{\sum_{\alpha,\beta\in\mathcal{E},\alpha\neq\beta}\sum_{i\in\alpha\cap\beta}|\alpha\cap\beta|-1}{Nk^2(g-1)},$$

$$\widehat{\theta'}_\circ = \frac{\sum_{\alpha,\beta\in\mathcal{E},\alpha\neq\beta}\sum_{i\in\alpha\cap\beta}|\alpha\cap\beta|-1}{Nk^2g},$$

where $0 \le \widetilde{\theta}_\circ \le 1-1/k$, $0 \le \widehat{\theta'}_\circ \le 1+1/(kg)-1/k-1/g$.

In such a homogeneous hypergraph, the critical synergy factor under GMC mechanism can be described as

$$r^*_{\mathrm{GMC}} = \frac{\frac{N}{g}-1}{\frac{N}{k}+\frac{N}{g}-\frac{N}{kg}+N\widehat{\theta}'_\circ-g} \tag{S118}$$

We quantify the impact of the hyperdegree on the evolutionary outcome under GMC mechanism by taking the partial derivative of the critical synergy factor with regard to the hyperdegree.

$$\frac{\partial r^*_{\mathrm{GMC}}}{\partial k} = \frac{N\left(1-\frac{N}{g}\right)^2}{k^2\left(\frac{N}{k}+\frac{N}{g}-\frac{N}{kg}+N\widehat{\theta}'_\circ-g\right)^2} > 0 \tag{S119}$$

Analogically, we take the partial derivative of the critical synergy factor with regard to the order to obtain how the order governs the evolutionary result of GMC mechanism.

$$\frac{\partial r^*_{\mathrm{GMC}}}{\partial g} = \frac{-kg^2+2kNg+(-N^2-k\widehat{\theta}'_\circ N^2-kN+N)}{kg^2\left(\frac{N}{k}+\frac{N}{g}-\frac{N}{kg}+N\widehat{\theta}'_\circ-g\right)^2} \tag{S120}$$

The above partial derivative will be negative when the number of the nodes is dominantly larger than the hyperdegree and the order.

As for HDB mechanism, the critical synergy factor on a homogeneous hypergraph can be described as

$$r^*_{\mathrm{HDB}} = \frac{N-2}{N+\frac{N}{k}+N\widetilde{\theta}_\circ-2g} \tag{S121}$$

Take the partial derivative of HDB's critical synergy factor with regard to the hyperdegree as

$$\frac{\partial r^*_{\mathrm{HDB}}}{\partial k} = \frac{N(N-2)}{k^2\left(N+\frac{N}{k}+N\widetilde{\theta}_\circ-2g\right)^2} > 0 \tag{S122}$$

Then take the partial derivative of HDB's critical synergy factor with regard to the order as

$$\frac{\partial r^*_{\text{HDB}}}{\partial g} = \frac{2(N-2)g}{\left(N + \dfrac{N}{k} + N\widetilde{\theta}_\circ - 2g\right)^2} > 0 \tag{S123}$$

Also in a homogeneous hypergraph, the critical synergy factor under HIM mechanism can be described as

$$r^*_{\text{HIM}} = \frac{N + \dfrac{N}{g} - 2}{N + \dfrac{N}{k} + \dfrac{N}{g} - \dfrac{N}{kg} + N\widehat{\theta}'_\circ - 2g}. \tag{S124}$$

We quantify the impact of the hyperdegree on the evolutionary outcome under HIM mechanism by taking the partial derivative of the critical synergy factor with regard to the hyperdegree.

$$\frac{\partial r^*_{\text{HIM}}}{\partial k} = \frac{\left(N + \dfrac{N}{g} - 2\right)\left(N - \dfrac{N}{k} - 2\right)}{g^2\left(N + \dfrac{N}{k} + \dfrac{N}{g} - \dfrac{N}{kg} + N\widehat{\theta}'_\circ - 2g\right)^2} > 0 \tag{S125}$$

Analogically, we take the partial derivative of the critical synergy factor with regard to the order to obtain how the order governs the result of HIM mechanism.

$$\frac{\partial r^*_{\text{HIM}}}{\partial g} = \frac{(2Nk-4k)g^2 + 4Nkg + (-N^2k\theta - 2N^2 - 2Nk + 2N)}{kg^2\left(N + \dfrac{N}{k} + \dfrac{N}{g} - \dfrac{N}{kg} + N\widehat{\theta}'_\circ - 2g\right)^2} \tag{S126}$$

We also derive that r^*_{HIM} reaches the minimum value when

$$g = \frac{-Nk + \sqrt{\Delta}}{Nk - 2k},$$

where $\Delta = N^2k^2 + (Nk-2k)(\frac{1}{2}N^2k\theta + N^2 + Nk - N)$.

7.2 Hyperdegree heterogeneity

To investigate how the hyperdegree heterogeneity influences the evolutionary outcomes, we adopt the hyperdegree-heterogeneous hypergraph where the nodes have distinct numbers of neighboring hyperedges and each hyperedge has the same order. Hence the topological parameters of such hypergraphs can be represented by $\langle g \rangle = g$, $\eta_k = 1$ and $\zeta = g\eta_k$. Also, we suppose that the hyperedgree heterogeneity does not affect the strength of overlaps between hyperedges.

On the hyperdegree-heterogeneous hypergraph, the critical synergy factor of GMC is therefore

$$r^*_{\text{GMC}} = \frac{\dfrac{N}{g} - \eta_k}{\dfrac{N}{\langle k \rangle} + N\widehat{\theta} - g\eta_k}. \tag{S127}$$

Taking the partial derivative of GMC's critical synergy factor [S127] with regard to the hyperdegree heterogeneity, η_k, as

$$\frac{\partial r^*_{\mathrm{GMC}}}{\partial \eta_k} = \frac{N\left(1-\widehat{\theta}-\dfrac{1}{\langle k\rangle}\right)}{\left(\dfrac{N}{\langle k\rangle}+N\widehat{\theta}-g\eta_k\right)^2} > 0. \tag{S128}$$

The critical synergy factor of the hyperdegree-heterogeneous hypergraph under HDB mechanism is

$$r^*_{\mathrm{HDB}} = \frac{N-2\eta_k}{N+\dfrac{N}{\langle k\rangle}+N\widetilde{\theta}-2g\eta_k}. \tag{S129}$$

By taking the partial derivative of HDB's critical synergy factor [S129] with regard to the hyperdegree heterogeneity, we have

$$\frac{\partial r^*_{\mathrm{HDB}}}{\partial \eta_k} = \frac{2N\left(g-1-\dfrac{1}{\langle k\rangle}-\widetilde{\theta}\right)}{\left(N+\dfrac{N}{\langle k\rangle}+N\widetilde{\theta}-2g\eta_k\right)^2} > 0. \tag{S130}$$

Then the critical synergy factor of the hyperdegree-heterogeneous hypergraph under HIM mechanism is

$$r^*_{\mathrm{HIM}} = \frac{N+\dfrac{N}{g}-2\eta_k}{N+\dfrac{N}{\langle k\rangle}+N\widehat{\theta}-2g\eta_k}. \tag{S131}$$

Similarly, take the partial derivative of HIM's critical synergy factor [S131] with the hyperdegree heterogeneity to obtain

$$\frac{\partial r^*_{\mathrm{HIM}}}{\partial \eta_k} = \frac{2N\left(g-\dfrac{1}{\langle k\rangle}-\widehat{\theta}\right)}{\left(N+\dfrac{N}{\langle k\rangle}+N\widehat{\theta}-2g\eta_k\right)^2} > 0. \tag{S132}$$

7.3 Order heterogeneity

We explore the impact of the order heterogeneity on the critical synergy factors under GMC, HDB and HIM using the order-heterogeneous hypergraph where the hyperedges hold the different orders. In the order-heterogeneous hypergraph, the topological parameters can be transformed into $\langle k\rangle = k$, $\eta_g = 1$ and $\zeta = \langle g\rangle\eta_g$. Also suppose that the order heterogeneity does not affect the overlap strength.

For the order-heterogeneous hypergraph, the critical synergy factor of GMC is thus

$$r^*_{\mathrm{GMC}} = \frac{\dfrac{N}{\langle g\rangle}-1}{\dfrac{N}{k}+N\widehat{\theta}-\langle g\rangle\eta_g}. \tag{S133}$$

Take the partial derivative of GMC's critical synergy factor Eq. S133 with regard to the order heterogeneity:

$$\frac{\partial r^*_{\mathrm{GMC}}}{\partial \eta_g} = \frac{N - \langle g \rangle}{\left(\frac{N}{k} + N\widehat{\theta} - \langle g \rangle \eta_g\right)^2} > 0. \tag{S134}$$

Under HDB mechanism, the critical synergy factor on the order-heterogeneous hypergraph is

$$r^*_{\mathrm{HDB}} = \frac{N-2}{N + \frac{N}{k} + N\widetilde{\theta} - 2\langle g \rangle \eta_g}. \tag{S135}$$

Take the partial derivative of HDB's critical synergy factor Eq. S135 with regard to the order heterogeneity:

$$\frac{\partial r^*_{\mathrm{HDB}}}{\partial \eta_g} = \frac{2\langle g \rangle (N-2)}{\left(N + \frac{N}{k} + N\widetilde{\theta} - 2\langle g \rangle \eta_g\right)^2} > 0. \tag{S136}$$

Finally, under HIM mechanism, the critical synergy factor on the order-heterogeneous hypergraph is

$$r^*_{\mathrm{HIM}} = \frac{N + \frac{N}{\langle g \rangle} - 2}{N + \frac{N}{k} + N\widehat{\theta} - 2\langle g \rangle \eta_g}. \tag{S137}$$

By taking the partial derivative of HIM's critical synergy factor Eq. S137 with regard to the order heterogeneity, we have

$$\frac{\partial r^*_{\mathrm{HIM}}}{\partial \eta_g} = \frac{2\langle g \rangle \left(N + \frac{N}{\langle g \rangle} - 2\right)}{\left(N + \frac{N}{k} + N\widehat{\theta} - 2\langle g \rangle \eta_g\right)^2} > 0. \tag{S138}$$

7.4 Overlap strength

For the homogeneous hypergraph, we define the strength of overlaps between hyperedges as

$$C_{\mathrm{ovl}} = \frac{\sum_{\alpha,\beta\in\mathcal{E},\alpha\neq\beta} |\alpha \cap \beta|^2}{Nk(k-1)g}. \tag{S139}$$

Substituting the above definition of the overlap strength, the critical synergy factor for the homogeneous hypergraph under GMC mechanism can be described as

$$r^*_{\mathrm{GMC}} = \frac{\frac{N}{g} - 1}{\frac{N}{k} + N\left(1 - \frac{1}{k}\right) C_{\mathrm{ovl}} - g}. \tag{S140}$$

The critical synergy factor for the homogeneous hypergraph under HDB mechanism can be described as

$$r^*_{\mathrm{HDB}} = \frac{N-2}{N + \frac{N}{k} + N\left(1 - \frac{1}{k}\right)\frac{gC_{\mathrm{ovl}} - 1}{g-1} - 2g} \tag{S141}$$

And under HIM mechanism, the critical synergy factor for the homogeneous hypergraphcan be described as

$$r^*_{\mathrm{HIM}} = \frac{N + \dfrac{N}{g} - 2}{N + \dfrac{N}{k} + N\left(1 - \dfrac{1}{k}\right) C_{\mathrm{ovl}} - 2g}. \tag{S142}$$

For all the mechanisms of GMC, HDB and HIM, the overlap strength can undoubtedly promote cooperation by decreasing the critical synergy factors.

8 Evolutionary game under pairwise updates

We refer to a previous work [2] to give the critical condition of PGGs under pairwise updates, applicable to any hypergraph. The hypergraph can be projected into the graph, preserving only the pair-wise connection relationships. The probability of the random walk on such a pairwise graph from i to j is described as p_{ij}.

8.1 Death-birth mechanism

The critical synergy factor of death-birth (DB) mechanism is

$$r^* = \frac{\sum_{i,j\in\mathcal{N}} \pi_i \sum_{e\in\mathcal{E}} p_{ij}^{(2)} \widetilde{\tau}_{ij}}{\sum_{i,j,y\in\mathcal{N}} \pi_i p_{ij}^{(2)} \sum_{e\in\mathcal{E}} \dfrac{b(j,e)b(y,e)}{k_j} \widetilde{\tau}_{iy} - \sum_{i,j\in\mathcal{N}} \pi_i \sum_{e\in\mathcal{E}} \dfrac{b(i,e)b(j,e)}{k_i} \widetilde{\tau}_{ij}} \tag{S143}$$

where $\widetilde{\tau}_{ij}$ represents the coalescence time with the node i and the node j, defined as

$$\widetilde{\tau}_{ij} = \begin{cases} 1 + \dfrac{1}{2}\left(\sum_{y\in\mathcal{N}} p_{iy}\widetilde{\tau}_{yj} + \sum_{y\in\mathcal{N}} p_{jy}\widetilde{\tau}_{iy}\right) & i \neq j \\ 0 & i = j \end{cases}. \tag{S144}$$

When the synergy factor exceeds this positive critical threshold, natural selection favors cooperation over defection under DB mechanism.

8.2 Pair-comparison mechanism

The critical synergy factor of pair-comparison (PC) mechanism is

$$r^* = \frac{\sum_{i,j\in\mathcal{N}} \pi_i \sum_{e\in\mathcal{E}} p_{ij} \widetilde{\tau}_{ij}}{\sum_{i,j,y\in\mathcal{N}} \pi_i p_{ij} \sum_{e\in\mathcal{E}} \dfrac{b(j,e)b(y,e)}{k_j} \widetilde{\tau}_{iy} - \sum_{i,j\in\mathcal{N}} \pi_i \sum_{e\in\mathcal{E}} \dfrac{b(i,e)b(j,e)}{k_i} \widetilde{\tau}_{ij}} \tag{S145}$$

Similarly, when the synergy factor exceeds this positive critical threshold, natural selection favors cooperation over defection under PC mechanism.

9 Example of specific structural population

9.1 Isolated population

We first calculate the evolutionary outcomes of an isolated population (Fig. S1a) under five specific mechanisms. An isolated population is just a well-mixed group with the node number $N = g$ and the hyperedge number $E = 1$. We fix the size of the hypergraph as $N = 100$. Thus the topological parameters of such a hypergraph can be represented as $\langle k \rangle = 1$, $\langle g \rangle = 100$, $\eta_k = 1$, $\zeta = 100$ and $\widetilde{\theta} = \widehat{\theta} = 0$. Then the critical synergy factor of the isolated population under GMC mechanism does not exist since both the numerator and the denominator equal zero. As for HDB and HIM mechanisms, their critical synergy factors of the isolated population approach infinity.

9.2 Overlapped population

We then investigate a population of three overlapping groups (Fig. S1b), on which the trait evolves under five specific mechanisms. Each group holds 100 nodes (*i.e.*, $g = 100$), and the intersection of each pair of groups includes just one node. Thus the topological parameters of the hypergraph become $N = 297$, $\langle k \rangle = \frac{100}{99}$, $\eta_k = \frac{499851}{490000}$, $\zeta = \frac{499851}{4900}$, $\widetilde{\theta} = 0$ and $\widehat{\theta} = \frac{99}{980000}$. Then the critical synergy factors of the overlapped population under GMC, HDB and HIM mechanisms can be calculated as

$$r^*_{\text{GMC}} = \frac{\frac{N}{g} - \eta_k}{\frac{N}{\langle k \rangle} + N\widehat{\theta} - g\eta_k} = \frac{\frac{297}{100} - \frac{499851}{490000}}{297 \cdot \frac{99}{100} + 297 \cdot \frac{99}{980000} - 100 \cdot \frac{499851}{490000}} \approx 0.01015, \tag{S146}$$

$$r^*_{\text{HDB}} = \frac{N - 2\eta_k}{N + \frac{N}{\langle k \rangle} + N\widetilde{\theta} - 2g\eta_k} = \frac{297 - 2 \cdot \frac{499851}{490000}}{297 + 297 \cdot \frac{99}{100} - 2 \times 100 \cdot \frac{499851}{490000}} \approx 0.75817, \tag{S147}$$

$$r^*_{\text{HIM}} = \frac{N + \frac{N}{g} - 2\eta_k}{N + \frac{N}{\langle k \rangle} + N\widehat{\theta} - 2g\eta_k} = \frac{297 + \frac{297}{100} - 2 \cdot \frac{499851}{490000}}{297 + 297 \cdot \frac{99}{100} + 297 \cdot \frac{99}{980000} - 2 \times 100 \cdot \frac{499851}{490000}} \approx 0.76574. \tag{S148}$$

10 Empirical network description

We analyze five empirical higher-order networks in this study, whose descriptions are as follows. The coauthorship network is derived from the computer science online bibliography DBLP, where each node indicates a scholar and each hyperedge contains all the scholars contributing to a publication. In the human contact network, each node is a student and each hyperedge is a group of students in close proximity during an interval. In the congress bill network, a node represents a congressperson and a hyperedge is comprised of the sponsor and co-sponsors of a bill. The email network is derived from a European institution, where a node is an email account and a hyperedge involves the sender and all recipients of an email. The online forum network is extracted from the forum Mathematics Stack Exchange, where each node is a user and each hyperedge represents a thread involving multiple users.

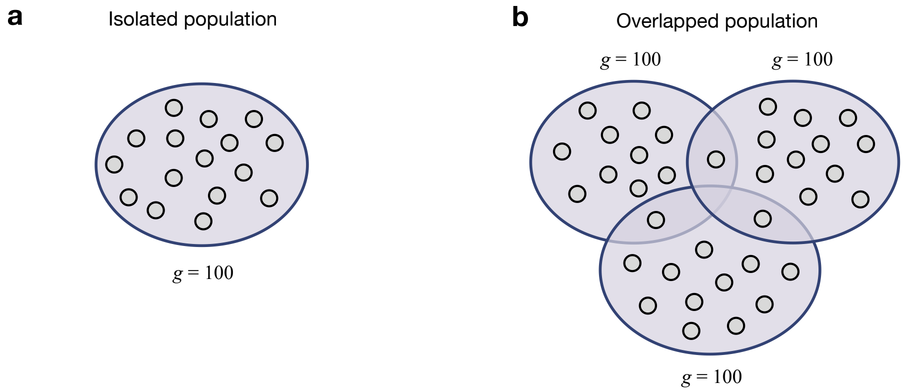

$r^*_{\mathrm{GMC}} \to \infty$ $\qquad$ $r^*_{\mathrm{GMC}} \approx 0.01015$

$r^*_{\mathrm{HDB}} \to \infty$ $\qquad$ $r^*_{\mathrm{HDB}} \approx 0.75817$

$r^*_{\mathrm{HIM}} \to \infty$ $\qquad$ $r^*_{\mathrm{HIM}} \approx 0.76574$

Figure S1: Isolated and overlapped populations

References

[1] Zhou, D., Huang, J. & Schölkopf, B. Learning with hypergraphs: Clustering, classification, and embedding. In *Advances in Neural Information Processing Systems*, 1601–1608 (2006).

[2] Allen, B. *et al.* Evolutionary dynamics on any population structure. *Nature* **544**, 227–230 (2017).

[3] Lawler, G. F. *Introduction to Stochastic Processes* (Chapman and Hall, New York, 2006), 2nd edn.

[4] Asavathiratham, C., Roy, S., Lesieutre, B. & Verghese, G. The influence model. *IEEE Control Systems Magazine* **21**, 52–64 (2001).

[5] Tan, S., Lü, J. & Hill, D. J. Towards a theoretical framework for analysis and intervention of random drift on general networks. *IEEE Transactions on Automatic Control* **60**, 576–581 (2015).
