# Supplementary material for: Emergence of cooperation promoted by higher-order strategy updates
Source: PLoS Comput Biol. 2025 Aug 4;21(8):e1012891. doi: 10.1371/journal.pcbi.1012891 (PMC12321138; doi:10.1371/journal.pcbi.1012891)
Supplement: S1 Text — (ZIP) [file pcbi.1012891.s001.zip › S1_Text/figure_s1.pdf]

a Isolated population

b Overlapped population

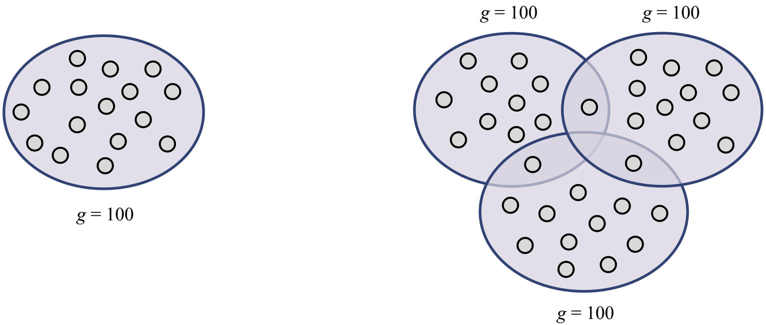

a

$$r^*_{\mathrm{GMC}} \to \infty$$

$$r^*_{\mathrm{HDB}} \to \infty$$

$$r^*_{\mathrm{HIM}} \to \infty$$

b

$$r^*_{\mathrm{GMC}} \approx 0.01015$$

$$r^*_{\mathrm{HDB}} \approx 0.75817$$

$$r^*_{\mathrm{HIM}} \approx 0.76574$$
