# Supplementary material for: Emergence of cooperation promoted by higher-order strategy updates
Source: PLoS Comput Biol. 2025 Aug 4;21(8):e1012891. doi: 10.1371/journal.pcbi.1012891 (PMC12321138; doi:10.1371/journal.pcbi.1012891)
Supplement: S1 Text — (ZIP) [file pcbi.1012891.s001.zip › S1_Text/figure_roadmap.pdf]

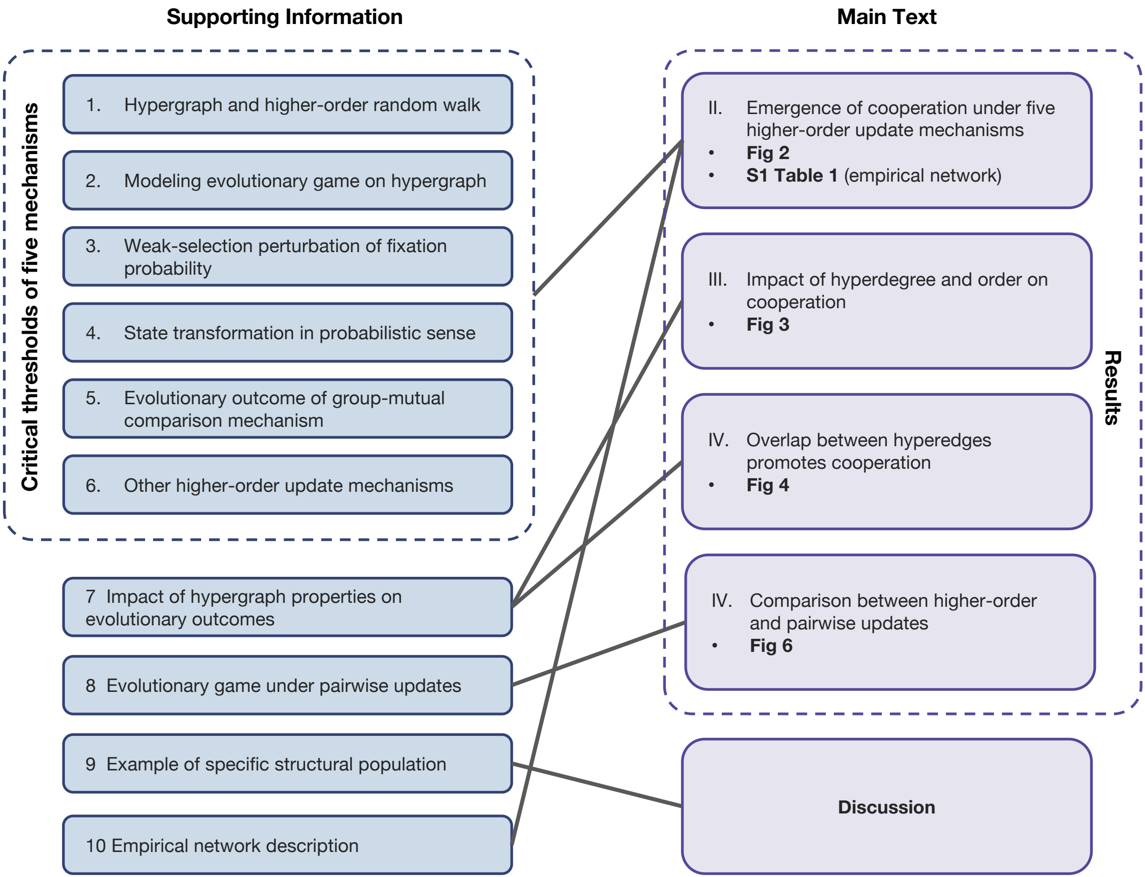
Supporting Information
Main Text
Critical thresholds of five mechanisms
1. Hypergraph and higher-order random walk
2. Modeling evolutionary game on hypergraph
3. Weak-selection perturbation of fixation probability
4. State transformation in probabilistic sense
5. Evolutionary outcome of group-mutual comparison mechanism
6. Other higher-order update mechanisms
7 Impact of hypergraph properties on evolutionary outcomes
8 Evolutionary game under pairwise updates
9 Example of specific structural population
10 Empirical network description
II. Emergence of cooperation under five higher-order update mechanisms
• Fig 2
• S1 Table 1 (empirical network)
III. Impact of hyperdegree and order on cooperation
• Fig 3
IV. Overlap between hyperedges promotes cooperation
• Fig 4
IV. Comparison between higher-order and pairwise updates
• Fig 6
Results
Discussion
