# Supplementary figures and images for: Emergence of cooperation promoted by higher-order strategy updates
Source: PLoS Comput Biol. 2025 Aug 4;21(8):e1012891. doi: 10.1371/journal.pcbi.1012891 (PMC12321138; doi:10.1371/journal.pcbi.1012891)

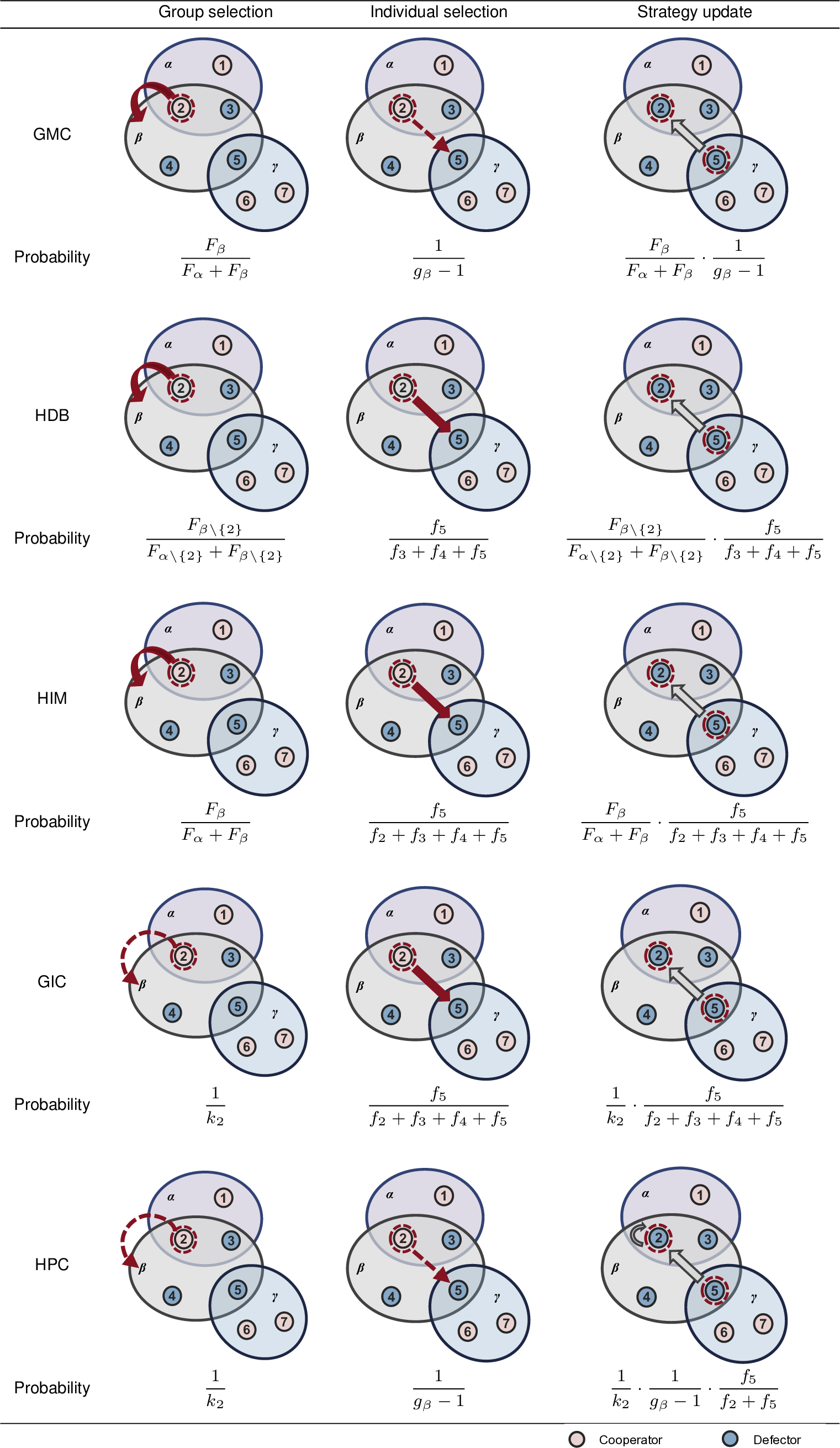

Supplement: S1 Fig — (TIF) [file pcbi.1012891.s002.tif]

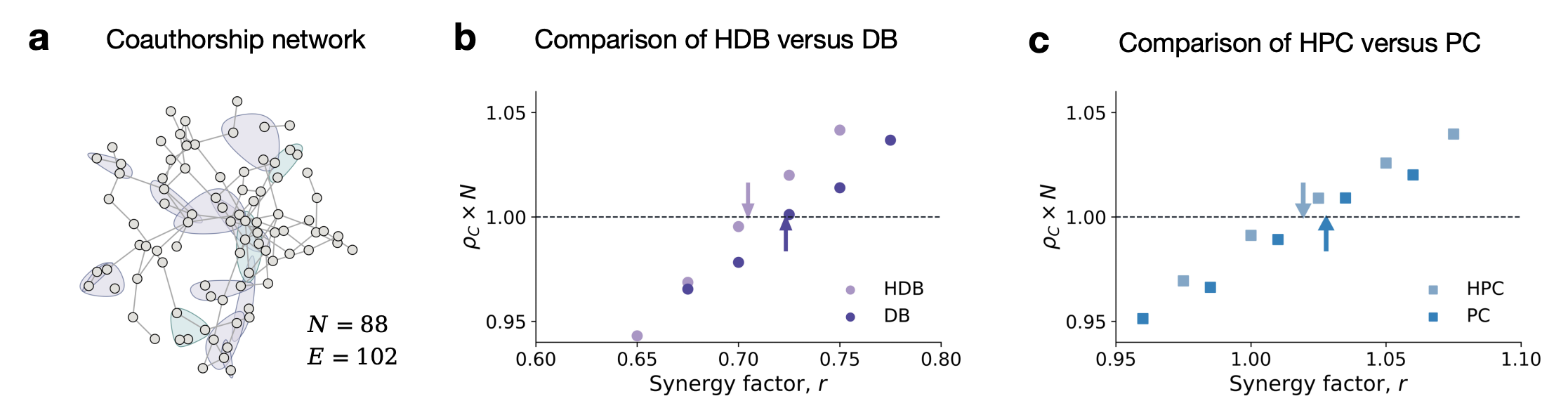

Supplement: S2 Fig — (TIF) [file pcbi.1012891.s003.tif]

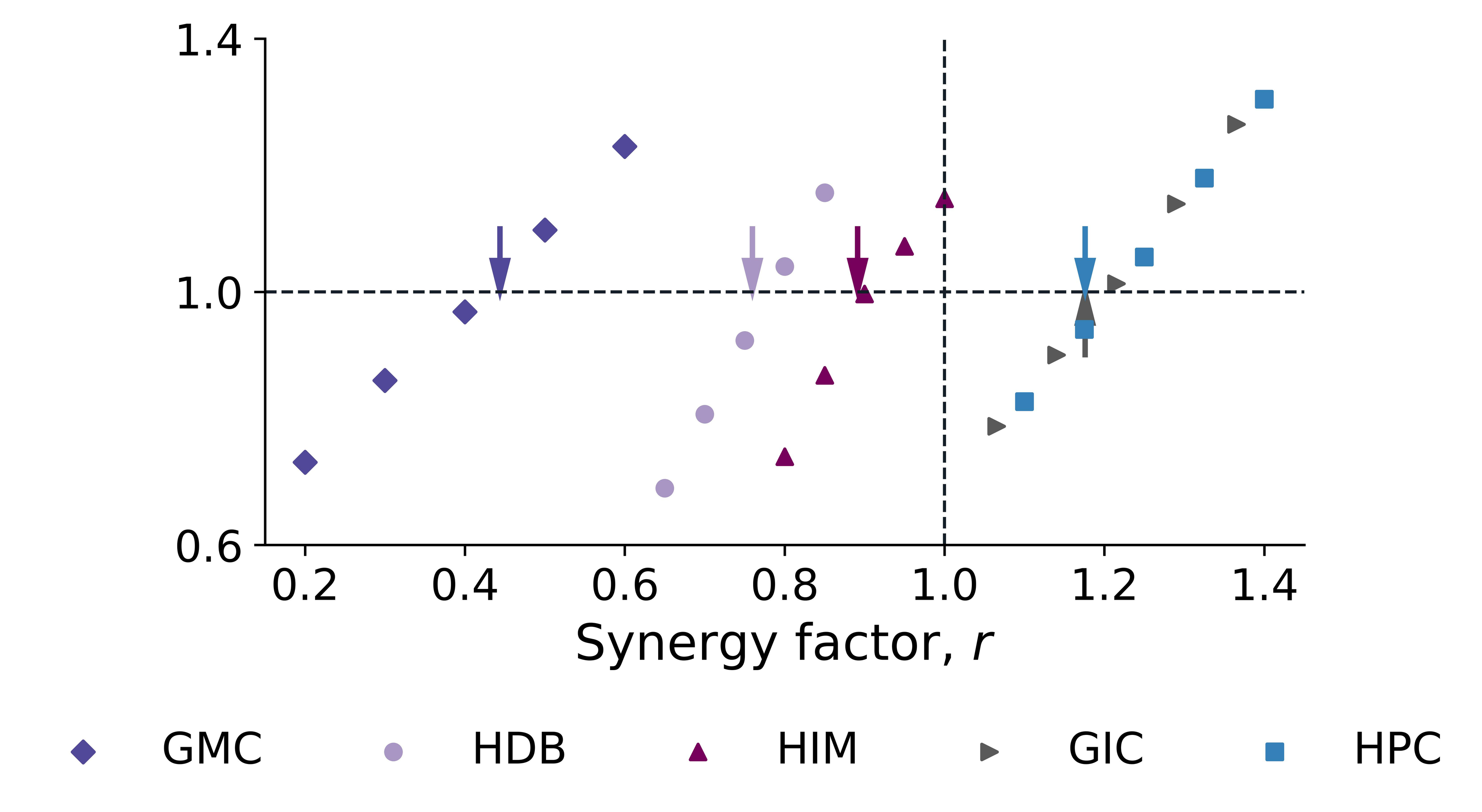

Supplement: S3 Fig — The population structure is inherited from Fig 2d as a hyperdegree-and-order-heterogeneous hypergraph. We set the intensity of selection as 0.2 and replicate each fixation process for 5×106 times. We can see a relative alignment between the simulation results (represented as scatters) and the analytical results of weak selection (indicated as arrows) under five update mechanisms. (TIF) [file pcbi.1012891.s004.tif]

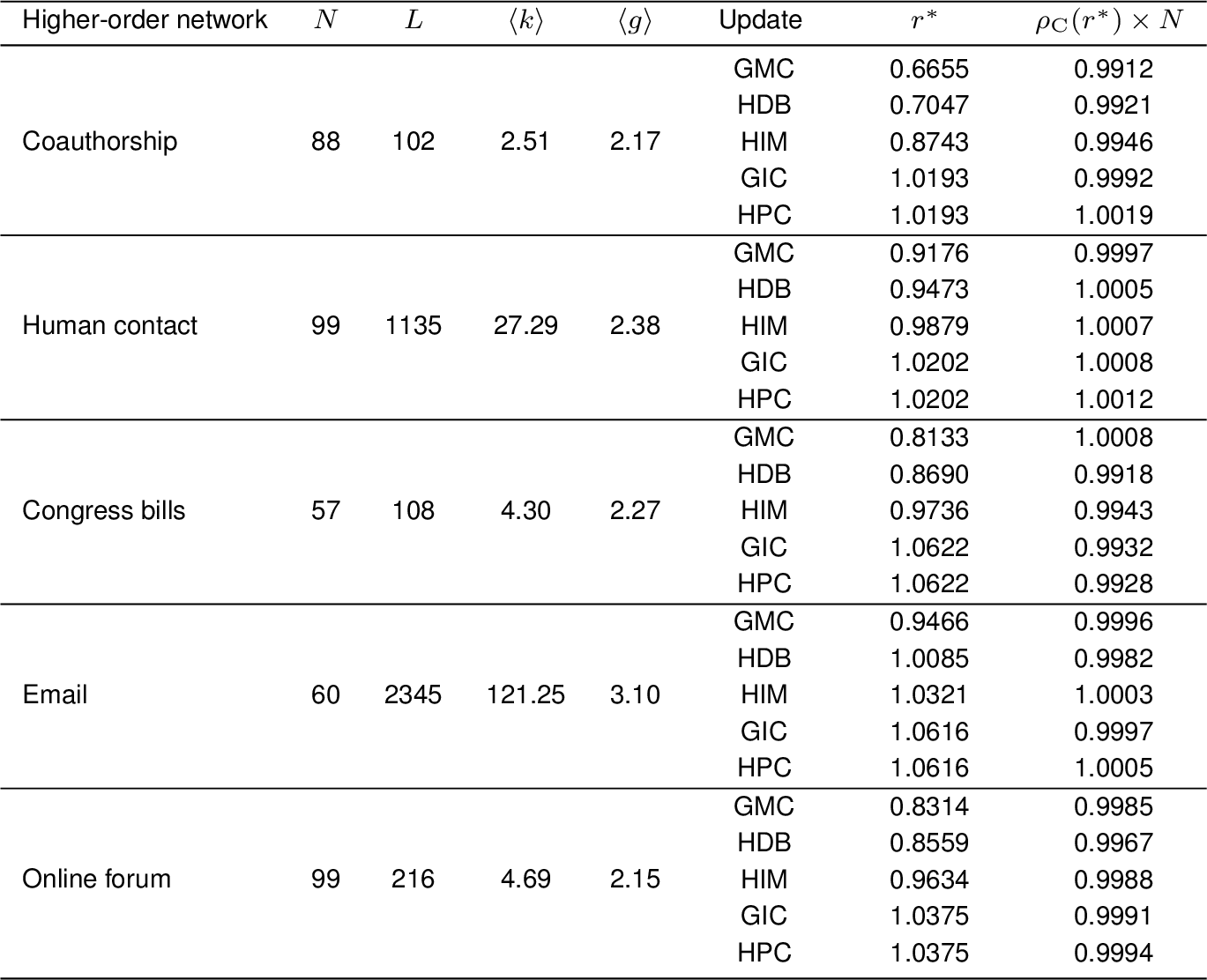

Supplement: S1 Table — The properties of five empirical higher-order networks [29]. Here the values of r* are our mathematical results for the critical synergy factors, and the last column shows the fixation probability times population size out of 5×10^5^ independent simulations under weak selection δ=0.025. Here, ρC(r*)×N=1.0 indicates a perfect agreement between our theoretical results and numerical simulations. (TIF) [file pcbi.1012891.s005.tif]
